# Anti-BCMA chimeric antigen receptors with fully human heavy-chain-only antigen recognition domains

**DOI:** 10.1038/s41467-019-14119-9

**Published:** 2020-01-15

**Authors:** Norris Lam, Nathan D. Trinklein, Benjamin Buelow, George H. Patterson, Namrata Ojha, James N. Kochenderfer

**Affiliations:** 1 National Institutes of Health, National Cancer Institute, Center for Cancer Research, Surgery Branch, NIH Building 10 Room 3-3888, Bethesda, MD 20892 USA; 2 TeneoBio, Inc. 7999 Gateway Blvd, Newark, CA 94560 USA; 3 National Institutes of Health, National Institute of Biomedical Imaging and Bioengineering, Section on Biophotonics. NIH Building 13 Room 3E33 13 South Drive, Bethesda, MD 20892 USA

**Keywords:** Expression systems, Cancer immunotherapy, T cells, Targeted therapies, Myeloma

## Abstract

Chimeric antigen receptor (CAR)-expressing T cells targeting B-cell maturation antigen (BCMA) have activity against multiple myeloma, but improvements in anti-BCMA CARs are needed. We demonstrated recipient anti-CAR T-cell responses against a murine single-chain variable fragment (scFv) used clinically in anti-BCMA CARs. To bypass potential anti-CAR immunogenicity and to reduce CAR binding domain size, here we designed CARs with antigen-recognition domains consisting of only a fully human heavy-chain variable domain without a light-chain domain. A CAR designated FHVH33-CD8BBZ contains a fully human heavy-chain variable domain (FHVH) plus 4-1BB and CD3ζ domains. T cells expressing FHVH33-CD8BBZ exhibit similar cytokine release, degranulation, and mouse tumor eradication as a CAR that is identical except for substitution of a scFv for FHVH33. Inclusion of 4-1BB is critical for reducing activation-induced cell death and promoting survival of T cells expressing FHVH33-containing CARs. Our results indicate that heavy-chain-only anti-BCMA CARs are suitable for evaluation in a clinical trial.

## Abstract (web summary)

Chimeric antigen receptors (CAR) use antibody variable regions to activate anti-tumor immunity. Here the authors show that a mouse IgH/IgL variable region used in a clinical CAR induces host immune responses to possibly reduce therapy efficacy, but an IgH-only CAR T design achieves similar CAR T activity but is potentially less immunogenic.

## Introduction

Chimeric antigen receptors (CAR) are artificial proteins that include antigen-recognition moieties, T-cell activation domains such as CD3ζ, and costimulatory domains such as 4-1BB and CD28^1–8^. B-cell maturation antigen (BCMA) is expressed by normal and malignant plasma cells and a small subset of B cells^9–11^. This restricted expression pattern makes BCMA a good target antigen for immunotherapies.

Multiple myeloma is an almost always incurable malignancy of plasma cells^12–15^. We and others have used CAR T cells targeting BCMA to treat multiple myeloma in clinical trials^16–19^. These clinical trials showed that 81–88% of patients had objective anti-myeloma responses after the treatment with anti-BCMA CAR T cells, but most anti-myeloma responses were not permanent^17–19^. Clearly, improvements in CAR T-cell therapies for multiple myeloma are needed.

Recipient anti-CAR immune responses that might limit CAR T-cell survival have been demonstrated against CAR T cells in clinical trials^20–22^. The 11D5-3 single-chain variable fragment (scFv) that has been incorporated into CARs used in several clinical trials^16,17,19^ is derived from a murine monoclonal antibody^11^. One way to potentially reduce immunogenicity of CAR binding domains is to use human instead of murine sequences^23,24^. Another potential way to decrease immunogenicity of CARs is to simplify the structure of the CAR’s antigen-binding domain by using heavy-chain-only binding domains^25^.

Immunoglobulin-like molecules with antigen-binding domains made up of only heavy chains without light chains were first described in camelids and cartilaginous fish^25–28^. Binding domains made up of only a single immunoglobulin heavy-chain variable region domain have been reported to exhibit strong and specific antigen binding^25,29–33^. Unlike a scFv, single heavy-chain-only binding domains have no need for a potentially immunogenic linker to connect the heavy chain to the light chain. Because human heavy-chain-only antigen-recognition domains have no linker or light chain, two potentially immunogenic junctions that must be included in scFvs are eliminated^25^. The smaller size of a heavy-chain-only antigen-recognition domain might have steric advantages over the larger scFv in reaching certain small or partially hidden cell surface antigens^25^. The smaller size of heavy-chain-only antigen-recognition domains is also an advantage for expression from gene therapy vectors, since decreasing the size of transgenes increases the titer of viral gene therapy vectors^34^. Compared with designs that utilize two scFvs, using two heavy-chain-only antigen-recognition domains simplifies design of bispecific CAR constructs capable of recognizing two antigens^35,36^.

The advantages of reduced size and potentially reduced immunogenicity of heavy-chain-only binding domains compared with scFvs led us to design and test the novel heavy-chain-only anti-BCMA CARs. T cells expressing a CAR with a heavy-chain-only antigen-recognition domain exhibit BCMA-specific functions equivalent to those of T cells expressing a CAR with the clinically proven 11D5-3-derived scFv. Including a 4-1BB costimulatory domain is especially important for improving survival of T cells expressing heavy-chain-only CARs. These results indicate that heavy-chain-only binding domains might offer general advantages for CARs targeting antigens other than BCMA.

## Results

### Immunogenicity of murine scFv-containing CARs

**Fig. 1:**
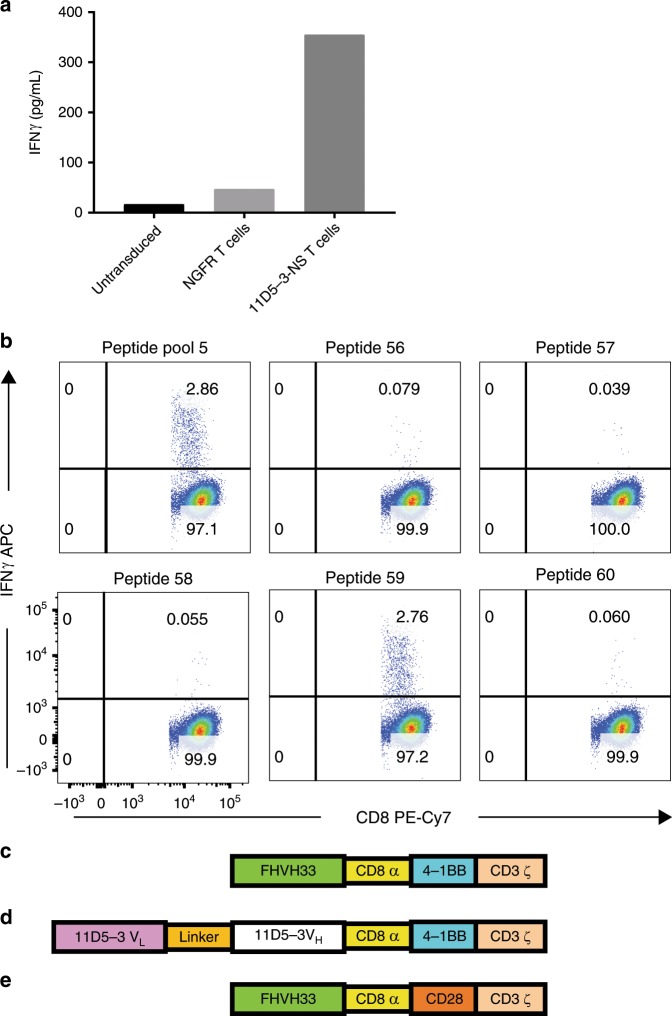
A murine anti-BCMA CAR can be immunogenic. **a** 11D5-3-NS, a truncated nonsignaling CAR containing only the murine 11D5-3 scFv, hinge, and transmembrane regions was designed. Irradiated, autologous 11D5-3-NS-transduced T cells were used to stimulate PBMC in culture. PBMC were from a patient who received 11D5-3-CD828Z CAR T cells on a clinical trial. Seven days later, the PBMC were stimulated again with 11D5-3-NS-transduced T cells. Seven days after the second stimulation, the PBMC were cultured overnight with autologous T cells that were either untransduced, transduced with the human NGFR gene, or transduced with the 11D5-3-NS gene. Culture supernatants were assayed for IFNγ by ELISA. 11D5-3-NS-specific release of IFNγ was found. **b** PBMC collected after CAR T-cell infusion to a different patient than in **a** were stimulated with autologous 11D5-3-CD828Z CAR^+^ T cells as in **a**. Peptide reactivity was assessed by culturing the stimulated PBMC for 6 h with autologous dendritic cells pulsed with 15-mer peptides of a peptide library covering all possible 15 mers of the 11D5-3 scFv. Specific IFNγ production by T cells was found in an ICCS assay against peptide pool 5 and peptide 59 from pool 5. **c** A diagram of the FHVH33-CD8BBZ CAR with the fully human heavy-chain binding domain FHVH33, hinge and transmembrane domains from human CD8α, a human 4-1BB domain, and a human CD3ζ domain. **d** 11D5-3-CD8BBZ has a murine scFv binding domain. Otherwise, 11D5-3-CD8BBZ has an identical sequence as FHVH33-CD8BBZ. **e** Except for substitution of CD28 for 4-1BB, FHVH33-CD828Z is identical to FHVH33-CD8BBZ.

We attempted to elicit T-cell immune responses against the murine 11D5-3 scFv by methods similar to those previously used by others^37,38^. We prepared a nonsignaling CAR called 11D5-3-NS. The extracellular and transmembrane sequences of 11D5-3-NS were identical to the corresponding sequences of 11D5-3-CD828Z, a CAR that has been previously tested in a clinical trial^17^. We transduced T cells from patients on the previous clinical trial of 11D5-3-CD828Z-expressing T cells^17^ with gamma-retroviral vectors encoding 11D5-3-NS (nine cases) or 11D5-3-CD828Z (one case). We used these transduced patient T cells as antigen-presenting cells to stimulate autologous peripheral blood mononuclear cells (PBMC) in culture. Two weekly stimulations of the PBMC with irradiated 11D5-3-expressing T cells were conducted. Next, we cultured the stimulated PBMC overnight with either autologous 11D5-3-expressing T cells or autologous negative-control T cells expressing the nerve growth factor receptor (NGFR) gene. We then performed an interferon gamma (IFNγ) enzyme-linked immunosorbent assay (ELISA) on the culture supernatants. We found specific PBMC reactivity against the 11D5-3-CAR in five out of ten patients evaluated. CAR-specific reactivity by the PBMC was defined as IFNγ release that was three-fold or more higher with 11D5-3-expressing T-cell targets compared with IFNγ release with negative-control NGFR-expressing T-cell targets. Another requirement for CAR-specific reactivity was a minimum of 150 pg/mL of IFNγ release by PBMC in response to 11D5-3-expressing T-cell targets (Fig. 1a).

To define the exact target of the anti-11D5-3 T-cell responses detected in the ELISA assays, we prepared 15-mer peptides that covered the whole sequence of the 11D5-3 scFv. We pulsed autologous dendritic cells with these peptides and used the peptide-pulsed dendritic cells to assay for 11D5-3-specific T cells among patient PBMC that had previously been stimulated two times in culture with autologous 11D5-3-expressing T cells. We detected peptide-specific immune responses by intracellular cytokine staining (ICCS) flow cytometry for IFNγ (Fig. 1b). We found 11D5-3-peptide-specific T cells in two out of two patients evaluated with this approach; this approach could only be used in two patients because an insufficient number of cells were available from other patients to conduct the assay. Peptides that elicited T-cell responses were from the complementarity determining region 3 plus framework 3 region of the 11D5-3 heavy chain in one patient and from the complementarity determining region 1 plus framework 1 region of the 11D5-3 light chain in the other patient.

### Design of CARs with heavy-chain-only binding domains

The potential for decreased immunogenicity of heavy-chain-only binding domains compared with scFvs and other advantages of CARs with heavy-chain-only binding domains led us to develop fully human heavy-chain-only CARs targeting BCMA. We previously reported novel rats transgenic for the complete functional human heavy-chain variable gene repertoire^30^. The rats are referred to as Unirats^30^. Unirats produce Uniabs that consist of two covalently linked antibody heavy chains. Unirats were immunized with human BCMA protein in adjuvant^30^. RNA was isolated from draining lymph nodes, and high-abundance heavy-chain variable region sequences were expressed as proteins^30,39^. These heavy-chain variable region domains were tested for BCMA recognition^30,39^. Sequences of four heavy-chain variable domains with the ability to specifically bind BCMA were used as antigen-recognition domains in novel CARs^40^. The CAR binding domains consisted of only a single fully human heavy-chain variable region. The four heavy-chain-only sequences incorporated into CARs were designated FHVH (fully human heavy-chain variable domain) 74, 32, 33, and 93^40^. In initial experiments, we constructed gamma-retroviral vectors encoding CARs that each contained one of the four heavy-chain-only domains. The CAR sequences started at the N-terminus with each FHVH domain followed by CD8α hinge and transmembrane domains, a 4-1BB costimulatory domain and a CD3ζ T-cell activation domain. A diagram of FHVH33-CD8BBZ is shown (Fig. 1c). T-cell surface expression of CARs containing FHVH74 was inferior to CARs containing the other three FHVH domains when 4-1BB was included in the CAR (Supplementary Fig. 1). CARs containing FHVH33, 32, and 93 were similar in expression and function, but FHVH33 was slightly superior in expression on T cells, so it was selected for further development. Supplementary Table 1 shows BCMA-specific IFNγ production by CARs with each FHVH domain.

We compared FHVH33-CD8BBZ to 11D5-3-CD8BBZ, a CAR that has the same sequence as FHVH33-CD8BBZ except the murine 11D5-3 scFv was included instead of FHVH33 (Fig. 1d). We also compared FHVH33-CD8BBZ to FHVH33-CD828Z that has the same sequence as FHVH33-CD8BBZ except for replacement of 4-1BB with CD28 (Fig. 1e).

### Expression and affinity of 11D5-3-CD8BBZ and FHVH33-CD8BBZ

**Fig. 2:**
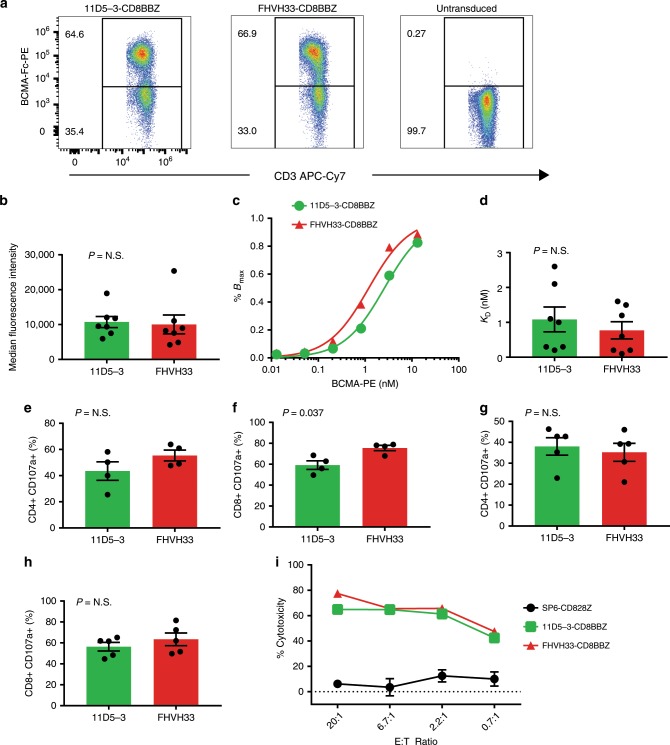
Murine scFv 11D5-3 versus fully human heavy-chain domain FHVH33. **a** Flow cytometry results of T cells from the same donor transduced with 11D5-3-CD8BBZ or FHVH33-CD8BBZ or left untransduced are shown; cells were stained with BCMA-Fc-PE. **b**, **d**–**h** show mean ± s.e.m. 11D5-3 means 11D5-3-CD8BBZ. FHVH33 means FHVH33-CD8BBZ. All comparisons are two-tailed, paired *t*-tests. *P* < 0.05 was considered statistically significant. N.S. is not statistically significant. **b** Median fluorescence intensity of CD3^+^, BCMA-Fc-PE^+^ cells expressing 11D5-3-CD8BBZ or FHVH33-CD8BBZ is shown (*n* = 7, *P* = N.S.). **c** Relative affinity of 11D5-3-CD8BBZ versus FHVH33-CD8BBZ was determined by staining CAR-expressing T cells with decreasing concentrations of BCMA-Fc-PE and performing flow cytometry. *Y*-axis is percent maximum specific binding (% of Bmax). **d** Relative *K*_D_ values were determined by nonlinear regression from binding curves of 11D5-3-CD8BBZ-expressing T cells and FHVH33-CD8BBZ-expressing T cells. *K*_D_ values were calculated based on the concentration of BCMA-Fc-PE yielding half-maximal binding (*n* = 7; *P* = N.S). T cells were transduced with either 11D5-3-CD8BBZ or FHVH33-CD8BBZ and stimulated in culture for 4 h; degranulation of CD4^+^ and CD8^+^ T cells was assessed by CD107a expression. T cells were stimulated with either BCMA^+^ C17-BCMA^−^K562 cells (**e** and **f**, *n* = 4) or BCMA^+^ RPMI8226 cells (**g** and **h**, *n* = 5). CD4^+^ or CD8^+^ %CD107a^+^ events are from flow cytometry plots gated on live CD3^+^ cells. Background degranulation with BCMA-negative NGFR-K562 cell stimulation was subtracted from degranulation with BCMA^+^ cell stimulation. Results were normalized for CAR expression. **i** T cells expressing 11D5-3-CD8BBZ, FHVH33-CD8BBZ, or negative-control CAR SP6-CD828Z were tested in a 4-h cytotoxicity assay (one of similar experiments). Points represent mean cytotoxicity of replicate wells ±s.e.m.

We used a BCMA-immunoglobulin fragment crystallizable (Fc)-phycoerythrin (PE) reagent to assess CAR expression on the surface of T cells. The percentages of T cells expressing 11D5-3-CD8BBZ versus FHVH33-CD8BBZ were not different 5 days after transduction (Fig. 2a). The median %CAR^+^ T cells was 57.2% (range 33.2–71.4%) for 11D5-3-CD8BBZ and 61.9% (range 49.0–74.3%) for FHVH33-CD8BBZ (*n* = 6; *P* = not significant, N.S.). Among CAR^+^ T cells after optimal BCMA-Fc-PE staining, there was no difference in median fluorescence intensity (MFI) between 11D5-3-CD8BBZ and FHVH33-CD8BBZ (Fig. 2b). We compared the relative affinity of 11D5-3-CD8BBZ and FHVH33-CD8BBZ by using methods adapted from previous work of others (Fig. 2c)^41,42^. We calculated dissociation constants (*K*_D_) by nonlinear regression. We found no difference in the *K*_D_ of 11D5-3-CD8BBZ versus FHVH33-CD8BBZ (Fig. 2d).

### Function of 11D5-3-CD8BBZ T cells versus FHVH33-CD8BBZ T cells

We did not find a difference in degranulation between 11D5-3-CD8BBZ and FHVH33-CD8BBZ except a small difference in CD8^+^ T-cell degranulation, when T cells were assayed with one out of two target cell lines tested (Fig. 2e–h). As with degranulation, we did not find a difference in cytotoxicity when 11D5-3-CD8BBZ and FHVH33-CD8BBZ were compared (Fig. 2i).

**Fig. 3:**
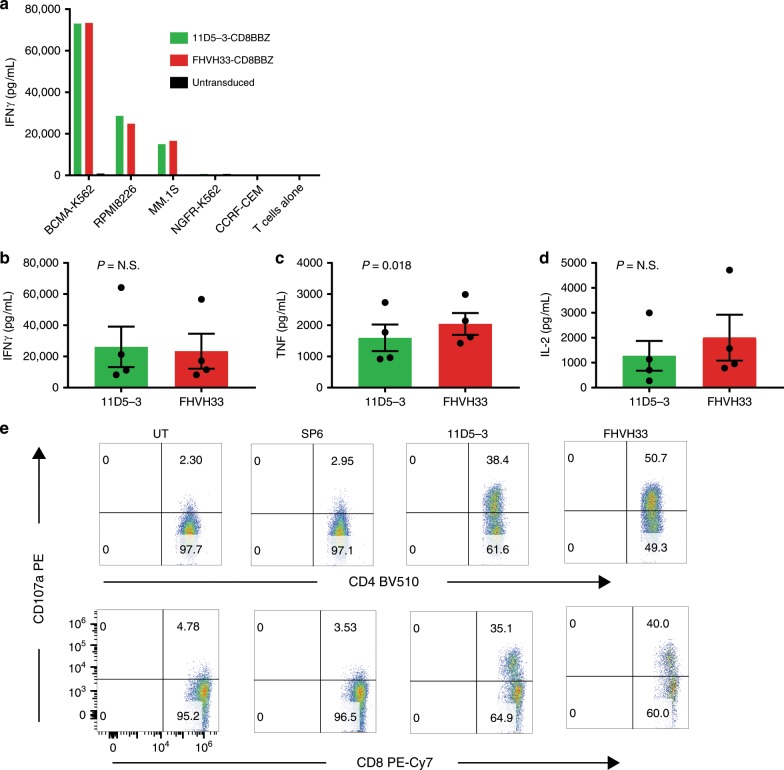
T cells expressing either 11D5-3-CD8BBZ or FHVH33-CD8BBZ specifically recognized BCMA and primary multiple myeloma cells. **a** T cells from the same donor were transduced with either 11D5-3-CD8BBZ or FHVH33-CD8BBZ or were left untransduced. T cells were cultured overnight with the indicated target cells, and an IFNγ ELISA was performed on the culture supernatant. Bars represent the means of duplicate wells. Bars representing cultures including untransduced T cells and cultures with BCMA-negative target cells are not visible because the values are too small. Similar results were obtained with cells from four different donors. BCMA-K562, RPMI8226, and MM.1 S are BCMA^+^; other target cells are BCMA^−^ (**b**–**d**) RPMI8226 target cells were cultured overnight with either 11D5-3-CD8BBZ (11D5-3) T cells or FHVH33-CD8BBZ (FHVH33) T cells. Next, an ELISA assay was performed to measure IFNγ, TNF, or IL-2 in the culture supernatant. The amount of nonspecific cytokine release in response to the BCMA-negative cell line NGFR-K562 in parallel cultures was subtracted from the cytokine release in response to RPMI8226 to obtain the RPMI8226-specific IFNγ release. Results are normalized for CAR expression. Mean  ±  s.e.m. are shown; *n* = 4. *P* = N.S. (not statistically significant) by paired two-tailed *t*-tests for IFNγ and IL-2; *P* = 0.018 for TNF. **e** Patient bone marrow cells that consisted of 56% BCMA^+^ multiple myeloma cells were cultured for 4 h with autologous T cells that were untransduced (UT) or transduced with the SP6-CD828Z negative-control CAR, 11D5-3-CD8BBZ, or FHVH33-CD8BBZ. Cells were stained for CD107a to detect degranulation of CD4^+^ T cells (top row) or CD8^+^ T cells (bottom row). Plots are gated on CD3^+^ live lymphocytes.

T cells expressing either 11D5-3-CD8BBZ or FHVH33-CD8BBZ specifically recognized BCMA^+^ target cell lines (Fig. 3a). The amounts of IFNγ and interleukin-(IL)-2 released by T cells expressing either CAR were not different; there was slightly higher tumor necrosis factor (TNF) production by FHVH33-CD8BBZ T cells (Fig. 3b–d). We quantified BCMA expression on target cell lines and assessed the ability of 11D5-3-CD8BBZ and FHVH33-CD8BBZ to recognize these cell lines by ICCS (Supplementary Fig. 2). In agreement with ELISA experiments, we found slightly higher TNF production by FHVH33-CD8BBZ T cells versus 11D5-3-CD8BBZ T cells. We assessed whether 11D5-3-CD8BBZ-expressing and FHVH33-CD8BBZ-expressing T cells could recognize primary human bone marrow multiple myeloma cells. We found that both CARs degranulated in response to the BCMA^+^ primary multiple myeloma cells (Fig. 3e). When T cells expressing 11D5-3-CD8BBZ or FHVH33-CD8BBZ were compared, there was no difference in BCMA-specific activation-induced cell death (AICD) or proliferation (Supplementary Figs. 3, 4). In contrast to the 4-1BB-containing CARs, when we compared CARs with CD28 domains in place of 4-1BB, we found higher levels of AICD with FHVH33-CD828Z versus 11D5-3-CD828Z (Supplementary Fig. 5). We did not observe a difference in aggregation of 11D5-3-CD828Z versus FHVH33-CD828Z CARs on the T-cell surface in experiments similar to previous work (Supplementary Fig. 6)^43^.

### Function of CARs with 4-1BB versus CD28 domains

**Fig. 4:**
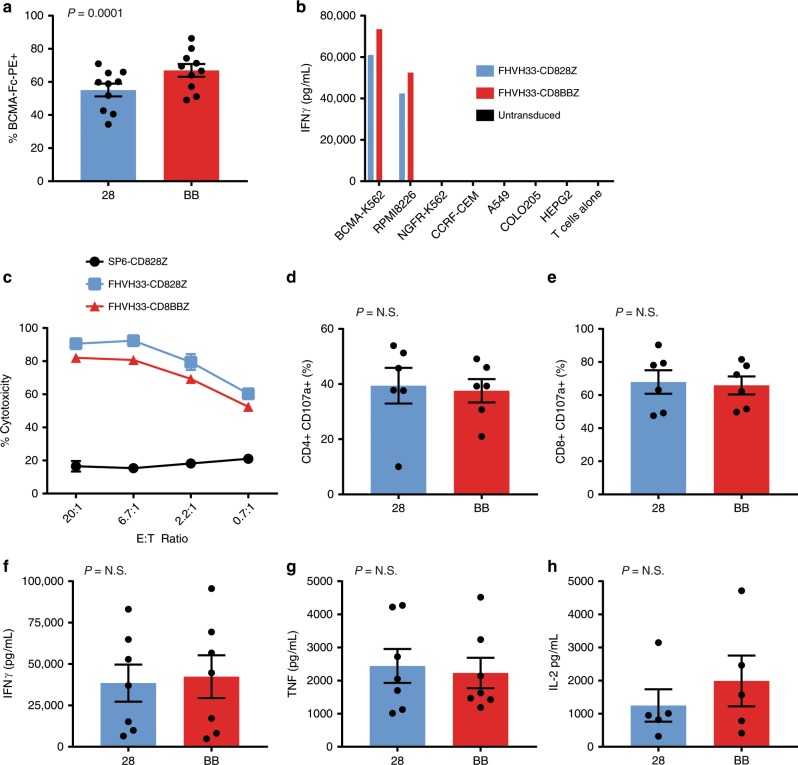
FHVH33 CARs with either a CD28 or 4-1BB costimulatory domain have similar in vitro function. **a** T cells from ten donors were transduced with either FHVH33-CD828Z (28) or FHVH33-CD8BBZ (BB). Four or 5 days after transduction, T cells were stained with anti-CD3 and BCMA-Fc-PE to detect CAR-expressing T cells. The mean ± s.e.m. of the percentages of T cells expressing each CAR is shown. All statistical comparisons in this figure were by paired two-tailed *t*-test. *P* < 0.05 was considered statistically significant. **b** T cells expressing either FHVH33-CD828Z or FHVH33-CD8BBZ or left untransduced were cultured overnight with the target cells indicated, and IFNγ was measured in the culture supernatant by ELISA. BCMA-K562 and RPMI8226 were BCMA^+^; the other cell lines were BCMA^−^. Bars represent the means of duplicate wells. The bars representing cultures including untransduced T cells and cultures with BCMA-negative target cells are not visible because the values are too small. Seven experiments with similar results were conducted. **c** T cells expressing either FHVH33-CD828Z, FHVH33-CD8BBZ, or the control CAR SP6-CD828Z were tested in a 4-h cytotoxicity assay. Two experiments with similar results were conducted. Symbols represent means of duplicate wells  ±s.e.m. Degranulation of CD4^+^ **d** or CD8^+^ **e** T cells was measured by detecting CD107a upregulation with flow cytometry (*n* = 6). The percentages of T cells with CD107a upregulation after a 4-h culture with RPMI8226 cells minus background CD107a upregulation after culture with NGFR-K562 cells are shown. Release of **f** IFNγ, **g** TNF, and **h** IL-2 after overnight culture with RPMI8226 cells minus background cytokine production after culture with NGFR-K562 cells is shown (*n* = 7 for IFNγ and TNF, *n* = 5 for IL-2). For **d**–**h**, the mean ± s.e.m. are shown; results are normalized for CAR expression; N.S, not statistically significant. *P* < 0.05 was considered statistically significant.

We performed a functional comparison of T cells expressing either FHVH33-CD8BBZ or FHVH33-CD828Z. The fraction of T cells expressing FHVH33-CD8BBZ was slightly higher than the fraction of T cells expressing FHVH33-CD828Z after transduction (Fig. 4a). We assessed CAR expression by the antibody binding capacity approach;^44^ we found similar levels of genomic integrations and CAR expression per genomic integration for T cells transduced with either FHVH33-CD828Z or FHVH33-CD8BBZ (Supplementary Fig. 7). We found that T cells expressing either FHVH33-CD8BBZ or FHVH33-CD828Z released IFNγ in a BCMA-specific manner (Fig. 4b). We found no difference in cytotoxicity (Fig. 4c) or degranulation (Fig. 4d, e) when FHVH33-CD8BBZ and FHVH33-CD828Z were compared. The quantities of IFNγ, TNF, and IL-2 released by CAR T cells expressing either FHVH33-CD8BBZ or FHVH33-CD828Z were not different (Fig. 4f–h). Because BCMA is a secreted protein that has been detected in the serum of multiple myeloma patients at a median level of 17.8 ng/mL^10^, we assessed whether solubilized BCMA could either block FHVH CAR recognition of BCMA^+^ target cells or cause nonspecific activation of CAR T cells. We found neither blocking of target cell recognition nor nonspecific CAR T-cell activation (Supplementary Table 2)

.

### Survival of T cells with FHVH33-CD8BBZ versus FHVH33-CD828Z

**Fig. 5:**
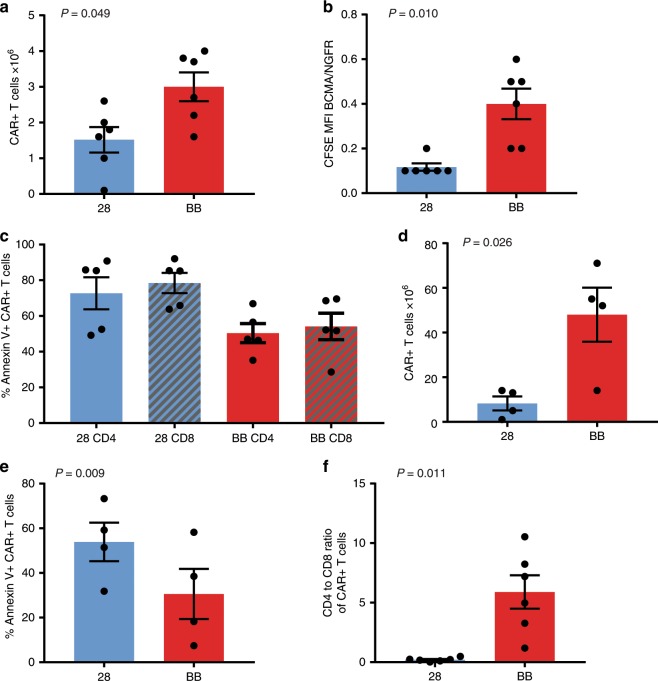
4-1BB versus CD28 CARs: proliferation and survival. **a** T cells expressing FHVH33-CD828Z (28) or FHVH33-CD8BBZ (BB) were labeled with CFSE and cultured with irradiated BCMA-K562 cells or NGFR-K562 cells. Changes in CAR^+^ T-cell numbers during the 4-day culture are shown (*n* = 6). All bar graphs in this figure show mean ± s.e.m.; all statistics are paired two-tailed *t*-tests, *P* < 0.05 was considered statistically significant. **b** BCMA-specific proliferation was represented by dividing the CFSE MFI of T cells stimulated with BCMA-K562 by the CFSE MFI of T cells stimulated with NGFR-K562. BCMA-specific CFSE dilution and proliferation were greater with FHVH33-CD828Z T cells (28) than FHVH33-CD8BBZ (BB) T cells (*n* = 6). **c** T cells expressing FHVH33-CD828Z (28) or FHVH33-CD8BBZ (BB) were cultured overnight with either BCMA-K562 cells or NGFR-K562 cells and stained with annexin V to detect apoptosis. As a measure of BCMA-specific apoptosis, the %annexin V^+^ CAR^+^ T cells was calculated as the percentage annexin V^+^ CAR^+^ T cells after BCMA-K562 stimulation minus the percentage annexin V^+^ CAR^+^ T cells after NGFR-K562 stimulation. The percentages of annexin V^+^ CAR^+^ cells were higher for FHVH33-CD828Z versus FHVH33-CD8BBZ for CD4^+^ (*P* = 0.017) and CD8^+^ T cells ^(^*P* = 0.007); *n* = 5. **d** T cells expressing FHVH33-CD828Z (28) or FHVH33-CD8BBZ (BB) were stimulated with BCMA-K562 cells. CAR^+^ T cells were quantified at the beginning of culture and 7 days later. Changes in CAR^+^ T-cell numbers between initiation and day 7 of culture are shown (*n* = 4). **e** After the culture described in **d**, T cells were stained for annexin V. Mean ± s.e.m. %annexin V^+^ CAR^+^ T cells is shown (*n* = 4). **f** Mean ± s.e.m. of CD4:CD8 ratios of CFSE-labeled 28 and BB CAR^+^ T cells after the 4-day culture from **a** are shown (*n* = 6).

We labeled T cells expressing either FHVH33-CD8BBZ or FHVH33-CD828Z with carboxyfluorescein succinimidyl ester (CFSE) and cultured them together with BCMA-K562 cells or negative-control NGFR-K562 cells. During a 4-day in vitro culture, the number of FHVH33-CD8BBZ CAR T cells increased more than the number of FHVH33-CD828Z T cells (Fig. 5a). We expressed BCMA-specific dilution of CFSE as the CAR T-cell CFSE MFI with BCMA-K562 stimulation divided by the CAR T-cell CFSE MFI with NGFR-K562 stimulation. A lower BCMA-K562/NGFR-K562 MFI ratio indicates greater CFSE dilution and more BCMA-specific proliferation. T cells expressing FHVH33-CD828Z proliferated more than T cells expressing FHVH33-CD8BBZ (Fig. 5b).

We hypothesized that the lesser accumulation of FHVH33-CD828Z T cells despite greater proliferation was due to increased T-cell death in FHVH33-CD828Z T cells relative to FHVH33-CD8BBZ T cells. We stimulated FHVH33-CD8BBZ and FHVH33-CD828Z T cells with BCMA-K562 (BCMA^+^) cells or BCMA-negative target cells overnight, and then we performed annexin V staining to detect apoptosis caused by AICD. We found higher AICD levels among T cells expressing FHVH33-CD828Z compared with T cells expressing FHVH33-CD8BBZ (Fig. 5c). We also found higher AICD levels with FHVH33-CD828Z versus FHVH33-CD8BBZ when RPMI8226 target cells were used in AICD assays (Supplementary Fig. 8). RPMI8226 cells express lower levels of BCMA compared with BCMA-K562 cells. With RPMI8226 target cells, levels of AICD were higher in CD8^+^ T cells than in CD4^+^ T cells regardless of which CAR was expressed; we did not see a difference in AICD between CD4^+^ and CD8^+^ T cells with BCMA-K562 target cells (Fig. 5c). There was no difference in AICD between T cell expressing either 11D5-3-CD828Z or 11D5-3-CD8BBZ when the T cells were cultured overnight with RPMI8226 target cells (Supplementary Fig. 9).

We next compared in vitro cell counts of FHVH33-CD8BBZ versus FHVH33-CD828Z T cells after 7 days of culture with BCMA-K562 cells. FHVH33-CD8BBZ T cells accumulated more than FHVH33-CD828Z T cells (Fig. 5d). We also detected lower levels of T-cell apoptosis as measured by annexin V staining of FHVH33-CD8BBZ T cells versus FHVH33-CD828Z T cells in this culture system (Fig. 5e). The cell surface phenotype of T cells at the start of the 7-day cultures in these experiments is shown in Supplementary Fig. 10. We also compared in vitro cell counts and apoptosis of 11D5-3-CD828Z T cells and 11D5-3-CD8BBZ T cells after 7 days of culture with BCMA-K562. Accumulation of T cells was higher and apoptosis was marginally lower for 11D5-3-CD8BBZ versus 11D5-3-CD828Z (Supplementary Fig. 11). In two series of culture experiments with BCMA-K562-stimulated T cells, we found a higher CD4 to CD8 ratio with FHVH33-CD8BBZ than with FHVH33-CD828Z (Fig. 5f, Supplementary Fig. 12).

### Murine studies of anti-BCMA CAR T cells

**Fig. 6:**
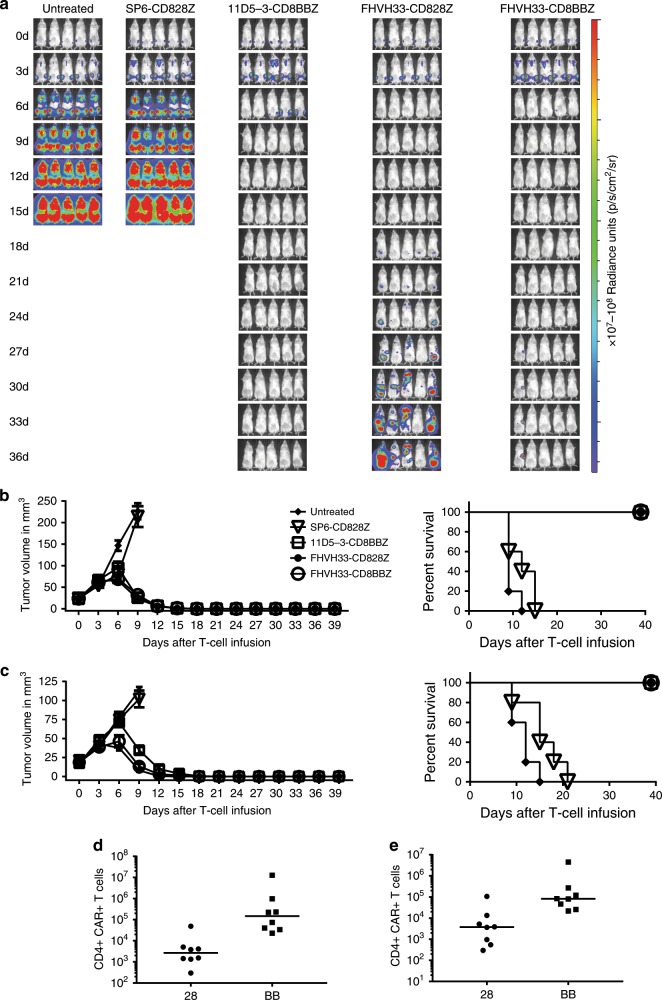
CAR T-cell studies in mice. **a** NSG mice were injected intravenously with MM.1 S cells. After 10 days, 1 × 10^6^ T cells expressing the indicated CARs were injected intravenously. Mice were imaged every 3 days. Two experiments of five mice each were completed with similar results with cells from different donors. **b**, **c** RPMI8226 cells were injected intradermally into NSG mice. After palpable tumors were established, mice were injected intravenously with **b** 2 × 10^6^ or **c** 1 × 10^6^ T cells expressing the indicated CARs. Left side graphs show the mean tumor volume of five mice/group. The right side graphs show Kaplan–Meier plots of survival of the same mice. For the 2 × 10^6^ CAR T-cell dose, there was a statistically significant difference in survival between the T cells expressing the SP6-CD828Z negative-control CAR and 11D5-3-CD8BBZ, FHVH33-CD828Z, and FHVH33-CD8BBZ (*P* = 0.003 for all three comparisons). For the 1 × 10^6^ CAR T-cell dose, there was a statistically significant difference in survival between the T cells expressing the SP6-CD828Z negative-control CAR and 11D5-3-CD8BBZ, FHVH33-CD828Z, and FHVH33-CD8BBZ (*P* = 0.002 for all three comparisons). Two experiments of five mice/group each were completed with T cells from different donors for each experiment with 2 × 10^6^ CAR^+^ T cells/mouse. **d** CD3^+^CD4^+^CAR^+^ and **e** CD3^+^CD8^+^CAR^+^ splenocytes were quantitated by flow cytometry 10 days after infusion of FHVH33-CD828Z (28) or FHVH33-CD8BBZ (BB) T cells. The NSG mice had disseminated MM.1 S tumor cells established prior to intravenous CAR T-cell infusion. Comparison by Mann–Whitney test. *P* = 0.0011 for the CD4 comparison, and *P* = 0.0030 for the CD8 comparison. Bars represent medians; *n* = 8 mice per group.

Disseminated malignancy burdens of the MM.1 S human multiple myeloma cell line were established in NOD.Cg-*Prkdc*^*scid*^ *Il2rg*^*tm1Wjl*^/SzJ (NSG) mice. The MM.1 S cells were reduced to below detectable levels by single injections of CAR T cells expressing each of the anti-BCMA CARs assessed: 11D5-3-CD8BBZ, FHVH33-CD828Z, and FHVH33-CD8BBZ; however, most mice receiving FHVH33-CD828Z-expressing T cells developed high-tumor-burden relapses (Fig. 6a, numerical bioluminescence comparisons are in Supplementary Fig. 13). We also evaluated anti-BCMA CAR T-cell therapy in mice bearing solid tumors of the RPMI8226 human myeloma cell line. A single intravenous injection of T cells expressing 11D5-3-CD8BBZ, FHVH-CD828Z, or FHVH33-CD8BBZ completely eliminated tumors at doses of 1 × 10^6^ CAR^+^ T cells/mouse (Fig. 6b) or 2 × 10^6^ CAR^+^ T cells/mouse (Fig. 6c). None of the anti-BCMA CARs exhibited anti-tumor activity at a dose of 0.5 × 10^6^ CAR T cells/mouse (Supplementary Fig. 14).

To evaluate persistence of CAR^+^ T cells, we established disseminated MM.1 S cells in NSG mice and injected T cells expressing either FHVH33-CD828Z or FHVH33-CD8BBZ. Ten days later, splenic CAR T cells were quantified. We found higher absolute numbers of FHVH33-CD8BBZ T cells than FHVH33-CD828Z T cells (Fig. 6d, e). Ratios of the median absolute number of CD4^+^CAR^+^ T cells to median absolute number of CD8^+^CAR^+^ T cells were calculated. The ratio for mice receiving FHVH33-CD8BBZ was 1.8, and the ratio for mice receiving FHVH33-CD828Z was 0.7.

Because MM.1 S cells were used in murine experiments, we also performed in vitro proliferation assays with the MM.1 S cells as BCMA^+^ target cells. MM.1 S expresses substantially lower levels of BCMA than the BCMA-K562 cells used in most of our in vitro proliferation assays (Supplementary Fig. 15). In vitro, we found greater accumulation of FHVH33-CD8BBZ versus FHVH33-CD828Z T cells for CD4^+^ T cells (Supplementary Fig. 16). There was also a trend toward greater accumulation of FHVH33-CD8BBZ versus FHVH33-CD828Z T cells for CD8^+^ T cells that did not reach statistical significance.

## Discussion

To address some of the limitations of CARs with scFv binding domains, we developed anti-BCMA CARs with antigen-binding domains consisting of only a single human heavy-chain variable region. There are three main rationales for using heavy-chain-only binding domains. First, the simpler structure of heavy-chain-only domains should be less immunogenic than an scFv that has an artificial linker and junctions between the light chain, linker, and heavy chain components. Our results demonstrate that T-cell responses can be elicited against the clinically used murine 11D5-3 scFv (Fig. 1). These results provide a rationale for developing less immunogenic CARs by using human rather than murine sequences and by using less complex antigen-binding domains, such as heavy-chain-only domains. Second, single heavy-chain-only domains are smaller than scFvs, and limiting the size of genes expressed by gene therapy vectors generally leads to the better gene expression by transduced T cells^34^. Limiting the size of expressed genes is especially important when more than one protein, such as two CARs need to be expressed^35,45,46^. Third, targeting multiple antigens simultaneously with the same CAR-expressing T cell is a major goal of the CAR field because of antigen loss from malignant cells targeted by CARs^35,45–49^. Heavy-chain-only binding domains ease design of bispecific CARs^35,36^. Targeting more than one antigen is important in multiple myeloma because some patients developed progressive multiple myeloma with loss of BCMA expression after anti-BCMA CAR T-cell therapy^16,17^.

We believe that our work is notable for the careful comparison that we have made to CARs with the 11D5-3 scFv, the binder of the CAR T-cell product that is the most advanced in clinical trials, bb2121. CARs incorporating 11D5-3 have been proven effective in two clinical trials^16,17,19^. When compared to CARs containing the clinically effective 11D5-3 scFv, the FHVH33-CD8BBZ CAR provides the advantages of fully human heavy-chain-only binding domains without any decrease of function of CAR-expressing T cells in vitro or in murine models.

We have initiated a phase I clinical trial of autologous T cells transduced with MSGV1-FHVH33-CD8BBZ. This is a dose-escalation trial that enrolls patients with relapsed or refractory multiple myeloma. The treatment protocol consists of a single infusion of CAR T cells preceded by cyclophosphamide and fludarabine conditioning chemotherapy. The primary objectives of the trial are to assess safety and feasibility of FHVH33-CD8BBZ T cells. Secondary objectives include assessment of the activity of FHVH33-CD8BBZ T cells against multiple myeloma and an assessment of in vivo proliferation and persistence of the CAR T cells.

CARs containing 4-1BB have different functional characteristics than CARs containing CD28, others have previously reported the ability of 4-1BB to increase T-cell persistence and resistance to AICD^2,8,50,51^. We have previously conducted extensive preclinical and clinical work with CARs containing CD28 costimulatory domains^11,16,17,23,52,53^. With traditional scFvs, we found that the CD28-containing CARs proliferated and survived well during in vitro culture^11,23,52^. In contrast to our previous work, in vitro survival of T cells expressing the CD28-containing CAR FHVH33-CD828Z was poor relative to the survival of T cells expressing the 4-1BB-containing CAR FHVH33-CD8BBZ. These observations were confirmed when we saw greater accumulation of FHVH33-CD8BBZ T cells compared with FHVH33-CD828Z T cells in two types of in vitro culture experiments (Fig. 5a, d). There was greater accumulation of FHVH33-CD8BBZ T cells despite the finding that FHVH33-CD828Z T cells proliferated more than FHVH33-CD8BBZ T cells when stimulated with BCMA^+^ target cells (Fig. 5b). We found more apoptosis in FHVH33-CD828Z versus FHVH33-CD8BBZ T cells after BCMA stimulation in three different experimental systems (Fig. 5c, e, and Supplemental Fig. 8); therefore, the likely explanation for lesser accumulation of FHVH33-CD828Z T cells relative to FHVH33-CD8BBZ T cells was increased death among FHVH33-CD828Z T cells versus FHVH33-CD8BBZ T cells. These in vitro findings were consistent with the lower numbers of FHVH33-CD828Z T cells versus FHVH33-CD8BBZ T cells persisting in mice 10 days after injection (Fig. 6d, e). Importantly, use of a 4-1BB costimulatory domain protected FHVH33 CARs from AICD; AICD levels were not different for CARs containing FHVH33 versus 11D5-3 when the CARs contained a 4-1BB costimulatory domains (Supplementary Fig. 3); however, when CARs with CD28 costimulatory domains were compared, T cells expressing FHVH33-CD828Z had higher levels of AICD compared with T cells expressing 11D5-3-CD828Z (Supplementary Fig. 5). 11D5-3-CD828Z had an identical sequence to FHVH33-CD828Z except for substitution of the 11D5-3 scFv for FHVH33. Interestingly, when we compared AICD in 11D5-3-CD828Z and 11D5-3-CD8BBZ, there was no statistically significant difference, which suggests that 4-1BB is more important in protecting against AICD with a CAR incorporating a heavy-chain-only binding domain versus a CAR with an scFv (Supplementary Fig. 9).

We consistently found a higher CD4 to CD8 ratio among FHVH33-CD8BBZ T cells versus FHVH33-CD828Z T cells (Fig. 5f, Supplemental Fig. 12). One possible factor contributing to the higher CD4 to CD8 ratio with FHVH33-CD8BBZ was that the mean ratio of CD4^+^ to CD8^+^ T-cell apoptosis was lower for FHVH33-CD8BBZ T cells than FHVH33-CD828Z T cells. (Supplementary Fig. 8). The difference in CD4^+^ to CD8^+^ ratio for FHVH33-CD8BBZ versus FHVH33-CD828Z was not due to differences in proliferation of CD8^+^ and CD4^+^ T cells (Supplementary Fig. 17). There was also a modestly higher CD4 to CD8 ratio of splenic CAR^+^ T cells in mice receiving FHVH33-CD8BBZ T cells versus mice receiving FHVH33-CD828Z T cells. Since the goal of our work is to produce an effective clinical CAR T-cell treatment, we are concerned about both the in vitro growth characteristics of CAR T cells and the in vivo properties of the T cells. Similar to predominance of CD4^+^CAR^+^ T cells in cultures of FHVH33-CD8BBZ T cells, there was a high CD4 to CD8 ratio of CAR^+^ T cells observed on a clinical trial of the bb2121 CAR T-cell product that has been clinically effective at treating multiple myeloma^19^. Like FHVH33-CD8BBZ, the bb2121 T-cell product has a CAR with a 4-1BB domain.

In summary, CARs with fully human heavy-chain-only binding domains have important advantages over scFv binding domains. The FHVH33 domain had no functional disadvantages in vitro or in murine models when compared to the 11D5-3 scFv, when a 4-1BB moiety is included in CARs.

## Methods

### Use of human cells and use of mice in experiments

PBMC and bone marrow cells were used from patients enrolled on National Cancer Institute (NCI) clinical trials. Use of patient samples for research was approved by the NCI Institutional Review Board. Informed consent was obtained from all patients. All animal studies were carried out on protocols approved by the NCI Animal Care and Use Committee.

### Gamma-retroviral transductions and T-cell culture

To produce replication-incompetent gamma-retroviruses, packaging cells were transfected with plasmids encoding CARs along with a plasmid encoding the RD114 envelope protein as previously described^52^. Gamma-retroviral transduction of T cells with genes encoding CARs or other proteins was performed as previously described 2 days after initiation of T-cell cultures^52^. T cells were cultured as described previously^52^. In brief, PBMC were stimulated with the anti-CD3 monoclonal antibody OKT3 (Ortho) in AIM V complete medium (Invitrogen) and 300 international units (IU)/mL of IL-2 (Teceleukin, Roche). AIM V complete medium consisted of AIM V medium (Thermo), 5% human AB serum (Valley Biomedical), 100 U/mL penicillin, and 100 μg/mL streptomycin. Non-tissue culture-treated six-well plates were coated with 10 μg/mL Retronectin (Takara). Two days after OKT3 stimulation, 2 mL of retroviral supernatant was applied to each well of the retronectin-coated, plates and incubated for 2 h at 37°C. Cells were resuspended in AIM V complete medium, and 2 × 10^6^ cells in 2 mL of medium were added directly to the viral supernatant. IL-2 was added to a final concentration of 300 IU/mL. Transduction was performed at 37 °C for 16–18 hours. Following transduction, cells were suspended in fresh AIM V complete media containing 300 IU/mL IL-2. Cultures were maintained by suspending the desired cell number to a concentration of 0.5 × 10^6^ cells/mL in AIM V complete media with 300 IU/mL IL-2 every 2 days.

### Generation of autologous stimulator cells

Autologous stimulator cells expressing a nonfunctioning version of the 11D5-3-CD828Z CAR were used to stimulate PBMC to induce proliferation of 11D5-3-specific T cells. The sequence of the 11D5-3-NS gene was as follows from N-terminus to C-terminus: the CD8α signal sequence, murine anti-BCMA scFv (11D5-3), and CD8α hinge and transmembrane sequence. The 11D5-3-NS sequence was synthesized as a gBlock fragment by Integrated DNA Technologies (IDT) and cloned into the MSGV1 gamma-retroviral vector backbone^54^ by using XhoI and BamHI restriction enzymes (NEB). Gamma-retroviral vector encoding 11D5-3-NS was produced and patient T cells were transduced as described under “Gamma-retroviral transductions and T-cell culture”. Patient T cells were also transduced with a gamma retroviruses made with a previously described plasmid^52^ encoding human full-length low-affinity NGFR. Cells were cryopreserved (Cryostor CS10, StemCell Technologies) for subsequent stimulations.

### Generation of a 15-mer peptide library from the 11D5-3 scFv

Individual 15-mer peptides with 11 amino acid overlapping sequences were generated against amino acids 8–279 of the 11D5-3-CD828Z CAR^11^ (PEPScreen, Sigma Aldrich). The 15-mers cover the light chain, linker, and heavy chain of the 11D5-3 scFv sequence. Individual peptides were dissolved in dimethyl sulfoxide to a concentration of 20 mg/mL. For rapid screening, 13 pools, each consisting of five peptides, were generated.

### Stimulation of PBMC with CAR-expressing stimulator cells

We cultured patient PBMC to assess for CAR-reactive T cells by modifying previously described methods^37,38^. In nine out of ten patients, patient PBMCs were stimulated with irradiated (3000 rad) autologous 11D5-3-NS-transduced stimulator T cells generated as described under “Generation of autologous stimulator cells”. In one patient, PBMC were stimulated with irradiated (3000 rad) autologous 11D5-3-CD828Z-transduced stimulator T cells because of insufficient starting cells to produce 11D5-3-NS stimulator cells.

CAR-reactive T cells were expanded using two different culture processes. Using the first method^37^, patient PBMC were thawed and cultured with irradiated autologous 11D5-3-NS stimulator cells at a 2:1 responder:stimulator ratio in T-cell complete media: RPMI1640 + L-glutamine (2 mM), heat-inactivated human AB serum (10%, Valley Biomedical), HEPES (25 mM, Corning), 2-mercaptoethanol (0.025 mM, Thermo Fisher), and Pen/Strep (100 U/mL penicillin, 100 mcg/mL streptomycin, Thermo Fisher). At day 3, IL-2 was added to the cultures (20 IU/mL). At day 7, responder PBMC were restimulated with irradiated 11D5-3-NS stimulator cells and irradiated autologous PBMC as feeder cells at a 2:1:4 responder:stimulator:feeder ratio. At days 9 and 11, IL-2 was added to the cultures (20 IU/mL). Using the second method^38^, patient PBMCs were thawed and cultured with irradiated autologous 11D5-3-NS stimulator cells at a 1:2 responder:stimulator ratio in T-cell complete media. At day 2, IL-2 was added to the cultures (300 IU/mL). At day 7, responder PBMC were restimulated with irradiated 11D5-3-NS stimulator cells at a 1:8 responder:stimulator ratio. At day 8, IL-2 was added to the cocultures (300 IU/mL). For both methods, day 14 responder PBMC were used for experiments to assess for reactivity to the 11D5-3 scFv.

### Assays to detect CAR-specific T-cell responses

For the detection of anti-11D5-3 scFv T-cell responses by IFNγ ELISA, day 14 responder PBMC were cultured alone or with autologous T cells expressing either 11D5-3-NS or NGFR at a 1:1 responder:stimulator ratio in T-cell complete media. Supernatants were assayed for IFNγ by ELISA after a 20-h incubation at 37°C.

For ICCS to assess stimulated PBMC reactivity with 15-mer peptide libraries, autologous monocyte-derived immature dendritic cells (MoDCs) were generated using methods previously described^55^. MoDCs were pulsed with 10 µg/mL of mixed peptides from peptide libraries or individual peptides overnight in T-cell complete media at 37°C. As a negative control, MoDCs were pulsed with 10 µg/mL hepatitis B core protein (HBC_128–140_) peptide. Responder PBMC were cultured with peptide-pulsed MoDCs at a 2:1 responder:stimulator ratio in the presence of no-azide/low-endotoxin-grade anti-CD28 and anti-CD49d antibodies (BD Biosciences) both at 1 µg/mL in T-cell complete media for 1 h at 37°C. After 1 h, Brefeldin A (GolgiPlug, BD Biosciences) was added (1:1000 dilution by the manufacturer’s instructions) and the cultures were incubated for an additional 5 h. Cells were stained for cell surface markers for CD3, CD4, and CD8, fixed with Cytofix/Cytoperm, and intracellular staining for IFNγ was conducted as recommended (BD Biosciences). Samples were analyzed by flow cytometry.

### Cell lines

RPMI8226 and MM.1 S are BCMA^+^ multiple myeloma cell lines that were obtained from ATCC. L363 is a BCMA^+^ myeloma cell line from DSMZ. A549 is a BCMA-negative lung cancer cell line (ATCC). CCRF-CEM is a BCMA-negative T-cell line (ATCC). HepG2 is a BCMA-negative hepatic carcinoma cell line (ATCC). Colo205 is a BCMA-negative colon carcinoma cell line (ATCC). BCMA-K562 are K562 cells (ATCC) transduced with the gene for full-length BCMA in our laboratory. C17-BCMA-K562 cells are BCMA-K562 cells cloned to limiting dilution and selected for low expression of BCMA. NGFR-K562 are K562 cells transduced with the gene for low-affinity nerve growth factor in our laboratory^52^. The same gamma-retroviral vector and methods were used to transduce BCMA-K562 and NGFR-K562. All cell lines were tested for mycoplasma and found to be negative.

### Design and construction of plasmids encoding FHVH CARs

We designed a series of CARs that contained fully human heavy-chain variable-region-only antigen-recognition domains (FHVH). The sequence of each CAR followed this pattern from the 5′-end to the 3′-end: CD8α signal sequence, one of 4 single FHVH domains, and the hinge and transmembrane regions of the human CD8α molecule. the cytoplasmic portion of either the CD28 or 4-1BB molecules, and the cytoplasmic portion of the CD3ζ molecule. The sequences used for CD8α, CD28, 4-1BB, and CD3ζ were obtained from the National Center for Biotechnology Information website (www.ncbi.nlm.nih). Guidance regarding the portions of each molecule to include in the CARs was obtained from prior work^52^.

The four fully human heavy-chain variable-region-only antigen-recognition domains were designated FHVH 74, 32, 33, and 93^40^. CARs were named in a systematic manner. For example, FHVH33-CD8BBZ has the FHVH33 antigen-recognition domain, a hinge and transmembrane region from CD8α, a 4-1BB costimulatory domain, and the CD3ζ T-cell activation domain.

All of these CARs were constructed and the CAR sequences were ligated into the MSGV1 gamma-retroviral vector backbone^54^ by standard methods. BCMA-specific variable heavy-chain sequences were synthesized as gBlocks by IDT. Each synthesized fragment consisted of a GTC trinucleotide, a NcoI site, the CD8α signal sequence, the FHVH sequence, part of the CD8α hinge and transmembrane domain, a BlpI site, and a TATCGT hexanucleotide. The GTC and TATCGT nucleotides were added to ensure complete end cleavage with NcoI and BlpI. Fragments were digested with BlpI and NcoI-HF (New England Biolabs) for 2 h at 37 °C. Digested fragments were then purified using the QIAquick PCR Purification kit (Qiagen). Fragments were ligated into the BlpI/NcoI-HF digested and gel-purified MSGV1 vector backbones that also included other components of the CAR not included in the gBlock fragments. The CAR components included in the MSGV1 vector backbones were: the remainder of the CD8α domain that was not included in the gBlock fragment, either CD28 or 4-1BB, and the CD3ζ domain. The ligation of each gBlock CAR fragment and the MSGV1 vector backbone fragment was carried out by using the Rapid DNA Ligation Kit (Roche Applied Science).

We also utilized the previously reported 11D5-3-CD828Z anti-BCMA CAR^11^, and 11D5-3-CD8BBZ, a CAR identical to 11D5-3-CD828Z except for replacement of CD28 by 4-1BB. The SP6 scFv recognizes the hapten 2, 4, 6-trinitrobenzenesulfonic acid^56^. We constructed a gamma-retroviral plasmid encoding a CAR with the SP6 scFv designated MSGV1-SP6-CD828Z, and we used it as a negative control.

### CAR detection on T cells and flow cytometry

T cells that were transduced with one of the CAR vectors and untransduced T cells were washed and stained with a BCMA-Fc protein labeled with PE (BCMA-Fc-PE) to detect cell-surface CAR molecules. T cells (5 × 10^5^) were suspended in 50 μL of staining buffer (0.4% bovine serum albumin, 0.1% sodium azide in phosphate-buffered saline; PBS), and a titrated amount of the BCMA-Fc-PE reagent (Creative Biomart) was added. For T-cell phenotyping experiments, cells were stained with the following antibodies: CD3 allophycocyanin (APC)-Cy7 (Clone UCHT1, BD Biosciences), CD4 FITC/BV510 (Clone RPA-T4, BD Biosciences; Biolegend), CD8 PE-Cy7/eFluor450 (Clone RPA-T8, BD Biosciences; Thermo Scientific), CD45RA FITC (Clone HI100, BD Thermo Scientific), CCR7 APC (Clone 150503, BD Biosciences), and CD57 FITC (Clone HCD57, Biolegend). For intracellular staining, cells were stained with IFNγ APC (Clone B27, BD Biosciences).

Flow cytometry was performed by standard methods. Dead cells were excluded by using 7-amino-actinomycin D (7-AAD, BD Biosciences). Flow cytometry analysis for all experiments was performed by using FlowJo (Tree Star, Inc.). The general flow cytometry gating strategies used are shown in Supplementary Fig. 18.

### Nonlinear regression analysis for CAR affinity

CAR affinity for BCMA protein was analyzed using the methods previously described^41,42^. A total of 5 × 10^5^ CAR T cells were stained for 30 minu at 37°C with BCMA-Fc-PE protein (Creative Biomart) diluted in four-fold serial dilutions; the cells were also stained with anti-CD3. For the first dilution, BCMA-Fc-PE protein was added to a final concentration of 13.175 nM in a 57.5 μL test volume. Flow cytometry was performed and the MFI of BCMA-Fc-PE-bound CAR T cells was determined. *K*_D_ values were calculated using the one-site binding (hyperbola) nonlinear regression model (GraphPad Prism 7) and the MFI of BCMA-Fc-PE-bound CAR T cells was expressed as a percentage of maximum binding sites (*B*_max_).

### CD107a assay

For each T-cell culture that was tested, two tubes were prepared. One tube contained C17-BCMA-K562 cells or RPMI8226 cells, and the other tube contained NGFR-K562 cells. Both tubes contained CAR-transduced T cells, 1 ml of AIM-V complete medium, a titrated concentration of an anti-CD107a antibody (Clone H4A3, Thermo Scientific), and 1 μL of Golgi Stop (monensin, BD). All tubes were incubated at 37 °C for 4 h and then stained for CD3, CD4, and CD8. Samples were analyzed by flow cytometry. Normalization was carried out by dividing the percentage of CD4^+^ or CD8^+^ T cells that were CD107a^+^ by the percentage of CD4^+^ or CD8^+^ T cells that were CAR^+^ by BCMA-Fc-PE staining.

### Cytotoxicity assay

Cytotoxicity assays were conducted as previously described^52,57^. Cytotoxicity was measured by comparing survival of RPMI8226 BCMA^+^ target cells relative to the survival of negative-control CCRF-CEM cells. Both of these cell types were combined in the same tubes with CAR-transduced T cells. CCRF-CEM negative-control cells were labeled with the fluorescent dye 5-(and-6)-(((4-chloromethyl)benzoyl)amino) tetramethylrhodamine (CMTMR; Thermo Scientific), and RPMI8226 BCMA^+^ target cells were labeled with labeled with carboxyfluorescein diacetate succinimidyl ester (CFSE; Thermo Scientific). In some experiments, CAR T-cell cultures were depleted of natural killer cells by flow cytometry (FACSAria, BD) prior to use in cytotoxicity assays. Cultures were set up in sterile 5 mL test tubes (BD) in duplicate at multiple T cell to target cell ratios. The target cells contained in the tubes were 50,000 CFSE-labeled RPMI8226 target cells along with 50,000 CMTMR-labeled CCRF-CEM negative-control cells. The cultures were incubated for 4 h at 37 °C. Immediately after the incubation, 7-AAD (BD Biosciences) was added, and flow cytometry acquisition was performed. For each T cell plus target-cell culture, the percent survival of RPMI8226 target cells was determined by dividing the percent live RPMI8226 cells by the percent live CCRF-CEM negative-control cells. The corrected percent survival of RPMI8226 target cells was calculated by dividing the percent survival of RPMI8226 target cells in each T cell plus target cell culture by the ratio of the percent live RPMI8226 target cells to percent live CCRF-CEM negative-control cells in tubes containing only RPMI8226 target cells and CCRF-CEM cells without effector T cells. This correction was necessary to account for variation in the starting cell numbers and for spontaneous target cell death. Cytotoxicity was calculated as follows: the percent cytotoxicity of RPMI8226 target cells = 100-corrected percent survival of RPMI8226 target cells.

### Annexin V staining

CAR-transduced T cells were incubated overnight in 24-well plates with either BCMA-K562, RPMI8226, or NGFR-K562 target cells with 1.5 × 10^6^ T cells and 1 × 10^6^ target cells in each well. After overnight incubation, cells were stained with BCMA-Fc-PE, CD3, CD4, and CD8. The cells were washed twice with PBS, resuspended in Annexin V Binding buffer (BD Biosciences), and incubated with APC-conjugated Annexin V (BD Biosciences) and 7-AAD (BD Biosciences) for 15 mins at room temperature. The cells were immediately analyzed by flow cytometry.

### Cytokine ELISA

BCMA^+^ or BCMA-negative target cells were combined with CAR-transduced T cells in duplicate wells of a 96-well round bottom plate in AIM-V complete medium at a 1:1 effector:target ratio. The plates were incubated at 37 °C for 18–20 h. Following the incubation, ELISAs for IFNγ were performed by using standard methods as previously described^23^. IL-2 and TNF ELISAs (R&D Systems) were performed as recommended by the manufacturer. When two or more CARs were compared, cytokine release was normalized for CAR expression by dividing the cytokine levels by the fraction of T cells expressing a given CAR.

### ICCS with BCMA^+^ cell lines

The %CAR^+^ cells within tested CAR T-cell cultures was normalized using autologous untransduced T cells (UT), such that the %CAR^+^ was the same for all tested CAR T-cell cultures. T cells were cocultured with BCMA^+^ cell lines at a 2:1 effector:target ratio in AIM V complete media without IL-2 for 6 h at 37°C in the presence of Brefeldin A (GolgiPlug, BD Biosciences, 1:1000 dilution by the manufacturer’s instructions). Cells were stained for CD3, fixed with Cytofix/Cytoperm, and intracellular staining for IFNγ, IL-2, and TNF was performed.

### Solubilized BCMA protein ELISA

Anti-BCMA CAR T cells were cultured either alone or with BCMA-K562 or RPMI8226 at a 1:1 effector:target ratio in AIM V complete media. Functional grade BCMA protein (Novus Biologicals, NBP2-34903) was added to the cultures to final concentrations of 25, 50, or 150 ng/mL. After 16 h at 37°C, IFNγ was assayed by ELISA of the culture supernatants as described^23^.

### Proliferation assays

Cultures were set up in 24-well plates. Target cells included in cultures were either 0.5 × 10^6^ irradiated BCMA-K562 cells or 0.5 × 10^6^ irradiated NGFR-K562 cells. In some experiments, MM.1 S replaced BCMA-K562 as the BCMA^+^ target. The cultures also included 0.75 × 10^6^ T cells from cultures that had been transduced with an anti-BCMA CAR. The T cells were labeled with CFSE (Thermo Scientific) as previously described^58^. The medium used in the cocultures was AIM V complete media. IL-2 was not added to the medium. Four days after initiation, the live cells in each culture were counted with trypan blue for dead cell exclusion, and flow cytometry was performed after staining with BCMA-Fc-PE, anti-CD3, anti-CD4, and anti-CD8. BCMA-specific proliferation presented as CFSE MFI of T cells stimulated with BCMA-K562 divided by the CFSE MFI of T cells stimulated with NGFR-K562.

### Antibody and BCMA-Fc-PE binding capacity measurements

Methods for the calculation of antibody or BCMA-Fc-PE binding capacity were based on the methods published in ref. ^44^. PE anti-human BCMA (FAB193P, R&D Systems) and BCMA-Fc-PE (Creative Biomart) were first titered on BCMA-K562 and FHVH33-CD8BBZ CAR T cells, respectively by using previously published methods^59^. PE antibody or BCMA-Fc-PE binding capacity was enumerated using BD Quantibrite™ PE beads using the manufacturer’s suggested protocol (Catalog: 340495, BD Biosciences).

### Vector copy number quantitative PCR

CD3^+^CAR^+^ FHVH33 T cells were sorted by flow cytometry using a BD FACSAria or BD Influx sorter. Genomic DNA from cells was extracted using the DNeasy Blood and Tissue Kit using the manufacturer’s suggested protocol (Qiagen). A transgene-specific quantitative PCR (qPCR) assay designed against the MSGV1 γ-retroviral vector was used.

RVV Forward: 5′-TTAGGTCACTGGAAAGATGTCG-3′

RVV Probe: 5′-6-FAM-AGACGTTGG-ZEN-GTTACCTTC-3′-IBFQ

RVV Reverse: 5′-GATGAGGTCTCGGTTAAAGGTG-3′

For a copy number reference, an RNaseP qPCR assay was used (catalog: 4403326, Thermo Fisher). For absolute quantification of vector copy number/cell, a calibrator plasmid was generated by cloning the human RNaseP gene sequence (NR_002312.1) into the MSGV1-FHVH33-CD8BBZ plasmid using BlpI and BamHI sites (New England Biolabs). For qPCR, the Quanta Perfecta Master Mix was used (catalog: 95076-012, Quanta) and reactions were cycled in a Roche LightCycler 96.

### Seven-day in vitro BCMA-specific culture assay

CAR T cells were cultured with irradiated BCMA-K562 target cells (18,000 rad) at a 2:1 effector:target ratio in AIM V complete media without IL-2 for 3 days. After 3 days, the total cells in culture were counted and AIM V complete media was added so that the final concentration of cells was 1 × 10^6^/mL. Initial and final CAR T cell numbers were calculated at the start and end of the 7-day culture period by counting cells with trypan blue for dead cell exclusion and performing flow cytometry with staining for BCMA-Fc-PE and anti-CD3. Cells at the end of the culture were also stained with Annexin V (BD Biosciences) to evaluate the percentage of apoptotic CAR^+^ T cells.

### Solid myeloma tumor in vivo experiments

NSG mice at 6–8 weeks of age from NCI-Frederick or the Jackson Laboratories were injected with 8 × 10^6^ RPMI8226 cells in PBS intradermally. Tumors were allowed to grow for 10 days until measurable tumors were present. CAR T cells that had been started in culture 7 days earlier were injected intravenously at doses ranging from 0.5 to 2 × 10^6^ CD3^+^CAR^+^ cells/mouse. Mice received one injection of CAR T cells. Tumors were measured using a caliper every 3 days, and the volume of the tumors were calculated using the formula (length × width × height)/2. Mice were sacrificed once tumors reached 15 mm in the longest length.

### Disseminated myeloma tumor experiments

MM.1 S cells were transduced in our lab with a retrovirus encoding an enhanced firefly luciferase gene^60^. MM.1S-luciferase cells were stained with a monoclonal antibody against Thy1.1 (BD Biosciences) and sorted by flow cytometry to purity (FACSAria, BD Biosciences). NSG mice from NCI-Frederick or the Jackson Laboratories were injected with 8 × 10^6^ MM.1S-luciferase cells intravenously. After 10 days, CAR T cells at a dose of 1–2 × 10^6^ CD3^+^CAR^+^ cells/mouse were infused intravenously. Mice received 1 injection of CAR T cells. For imaging, mice were injected with 100 µL of luciferin solution (15 mg/mL in PBS, GoldBio) and anesthetized with 3% isoflurane. After 10 min, bioluminescence imaging (BLI) was captured using a Xenogen IVIS Imaging System. Ventral images were taken using a 1-min exposure on a 24 cm field of view with a binning factor of 4. BLI was quantified over the body of the mouse without the tail in units of radiance (p/sec/cm^2^/sr) using the Living Image software (Xenogen). Images were scaled to 10^7^–10^8^ radiance units. Mice were sacrificed upon the onset of severe hind leg paralysis and wasting in accordance with the NCI Animal Care and Use Committee guidelines.

### In vivo T-cell persistence experiments

NSG mice (6–8 weeks old) from NCI-Frederick or the Jackson Laboratories were injected with 8 × 10^6^ MM.1S-luciferase cells in PBS intravenously. After 13 days, 1 × 10^6^ CD3^+^CAR^+^ T cells/mouse were infused intravenously. Mice were sacrificed at day 10 after T-cell infusion, and spleens were dissected from mice. Whole spleens were dissociated with a syringe plunger. Red blood cells were lysed with lysis buffer (Quality Biological) and 0.5 × 10^6^ splenocytes were used for flow cytometry detection of CAR T cells by staining with BCMA-Fc-PE, anti-CD3, anti-CD4, and anti-CD8. To calculate the number of CAR T cells/spleen, the total number of splenocytes per mouse was multiplied by the product of the %CD3^+^, %CD4^+^ or %CD8^+^, and the %CAR^+^.

### Dronpa imaging

BCMA CAR-Dronpa fusion proteins were generated by cloning the full-length Dronpa coding region to the 3′-ends of the 11D5-3-CD828Z and FHVH33-CD828Z coding regions in the MSGV1 γ-retroviral backbone.

The microscope used for imaging experiments is a home-built system on a Nikon TE2000 base described previously and reiterated here with necessary alterations^61^. The objective lens used for imaging was a Nikon 100 × /1.4 NA Oil Plan Apo. A 405 nm laser (LaserBoxx, Oxxius) was used to photoswitch proteins to the “on” state and a 488 nm laser (Sapphire, Coherent Inc.) was used to image Dronpa. Laser lines were combined using appropriate dichroic mirrors. The 405 nm laser current was controlled using the ESIo AOTF controller (ESImaging) and was shuttered using a diaphragm shutter with controller (part# SH025T, Thorlabs, Inc.) triggered using the ESIo AOTF controller (ESImaging). The 488 nm laser line was controlled using an AOTF (Gooch & Housego PLC). All lasers are passed through a linear polarizer (part# WP25M-VIS, Thorlabs) and directed toward objective using a 488 nm dichroic mirror (part# DiO3-R488, Semrock). The emission is passed through the dichroic and reflected toward camera using 45-degree mirror. Emission is passed through appropriate emission filters and passed through a Dual-View splitter (Photometrics). A DV2 POL cube (part# DV2-POL-CUBE-KIT, Photometrics) was inserted into the Dual-View imager which splits emission in orthogonal polarizations allowing a simultaneous recording of both images using a PCO Edge 4.2 LT (PCO AG) camera. The microscope was controlled using MicroManager. The estimated imaging power density was ~0.03 W/cm^2^ for 488 nm excitation.

### Statistics

The statistical tests used in each experiment are given in the figure legends of each figure. In general, we used two-tailed paired Student’s *t*-tests for analyzing in vitro experiments. Mouse survival curves were generated by the method of Kaplan and Meier. *P* values were not corrected for multiple comparisons. Mouse tumor measurements were blinded to the investigator performing the tumor measurements. In vitro assays were not blinded. GraphPad Prism 7 was used for statistical analysis and for making graphs. All ELISA and cytotoxicity assays were set up in duplicate wells. In all in vitro experiments, “*n*” refers to the number of independent experiments with cells from different donors. In murine experiments, “*n*” refers to an individual mouse. Values for “*n*” are given in the legends of each figure. Replicates of mouse adoptive T-cell transfer experiments were performed with T cells from different human donors. In all cases, *P* < 0.05 was considered statistically significant.

### Reporting summary

Further information on research design is available in the Nature Research Reporting Summary linked to this article.

## Supplementary information

Supplementarty Materials

Reporting Summary

## Data Availability

The authors will provide any primary data for results presented in this publication upon request. Upon reasonable request, we will provide materials not commercially available that were used in this work. GenBank accession codes of novel CARs reported in this manuscript were as follows: BankIt2257033 11D5-3-CD8BBZ MN366105 BankIt2257033 FHVH33-CD828Z MN366106 BankIt2257033 FHVH33-CD8BBZ MN366107

## Source data

Source data
