# Author Correction: Anti-BCMA chimeric antigen receptors with fully human heavy-chain-only antigen recognition domains

**DOI:** 10.1038/s41467-020-15145-8

**Published:** 2020-03-09

**Authors:** Norris Lam, Nathan D. Trinklein, Benjamin Buelow, George H. Patterson, Namrata Ojha, James N. Kochenderfer

**Affiliations:** 1 National Institutes of Health, National Cancer Institute, Center for Cancer Research, Surgery Branch, NIH Building 10 Room 3-3888, Bethesda, MD 20892 USA; 2 TeneoBio, Inc., 7999 Gateway Blvd, Newark, CA 94560 USA; 3 National Institutes of Health, National Institute of Biomedical Imaging and Bioengineering, Section on Biophotonics, NIH Building 13 Room 3E33 13 South Drive, Bethesda, MD 20892 USA

**Keywords:** Expression systems, Cancer immunotherapy, T cells, Targeted therapies, Myeloma

Correction to: *Nature Communications* 10.1038/s41467-019-14119-9, published online 15 January 2020.

In the original version of this Article, the Y axis label in Fig. 6, panel E was incorrectly labelled as CD4+ CAR+T cells, instead of CD8+ CAR+T cells. This error has been corrected in both the PDF and HTML versions of the Article.

