# Supplementary material for: Anti-BCMA chimeric antigen receptors with fully human heavy-chain-only antigen recognition domains
Source: Nat Commun. 2020 Jan 15;11:283. doi: 10.1038/s41467-019-14119-9 (PMC6962219; doi:10.1038/s41467-019-14119-9)
Supplement: Supplementary file 1 — Supplementarty Materials [file 41467_2019_14119_MOESM1_ESM.pdf]

Supplementary Information for:

Anti-B-cell maturation antigen (BCMA) chimeric antigen receptors (CARs) with fully-human heavy-chain-only antigen-recognition domains

Lam et al.

This file includes:

Supplementary Figures 1-18

Supplementary Tables 1-2

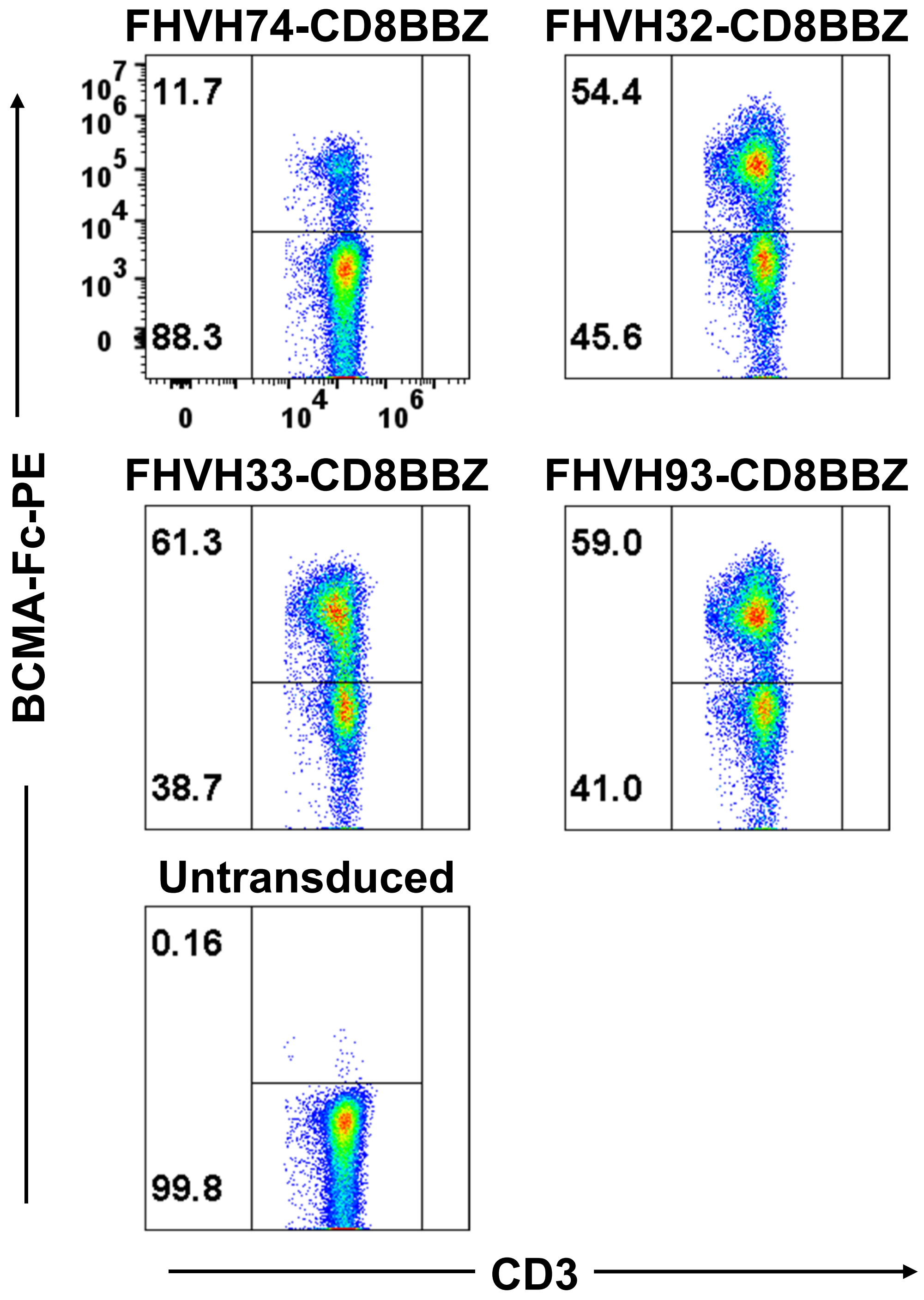

Supplementary Figure 1. Four anti-BCMA fully-human heavy-chain-only (FHVH) CARs were designed. Each CAR included a different FHVH binding domain (FHVH74, 32, 33, and 93), CD8α hinge and transmembrane domains, a 4-1BB costimulatory domain, and a CD3ζ T cell activation domain. T cells were transduced with γ-retroviruses encoding each CAR. Surface expression of CARs was evaluated 5 days after transduction by staining with BCMA-Fc-PE followed by flow cytometry. Cells were gated on $CD3^+$ events. Data shown are representative of two independent experiments using T cells derived from 2 different donors (n=2).

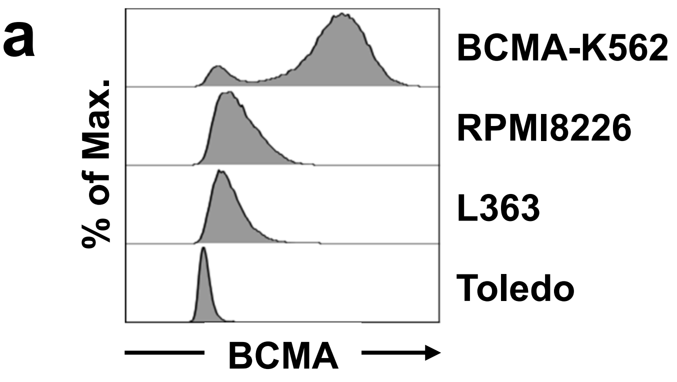

b

	PE anti-BCMA ABC
BCMA-K562	34899
RPMI8226	3554
L363	2724
Toledo	709

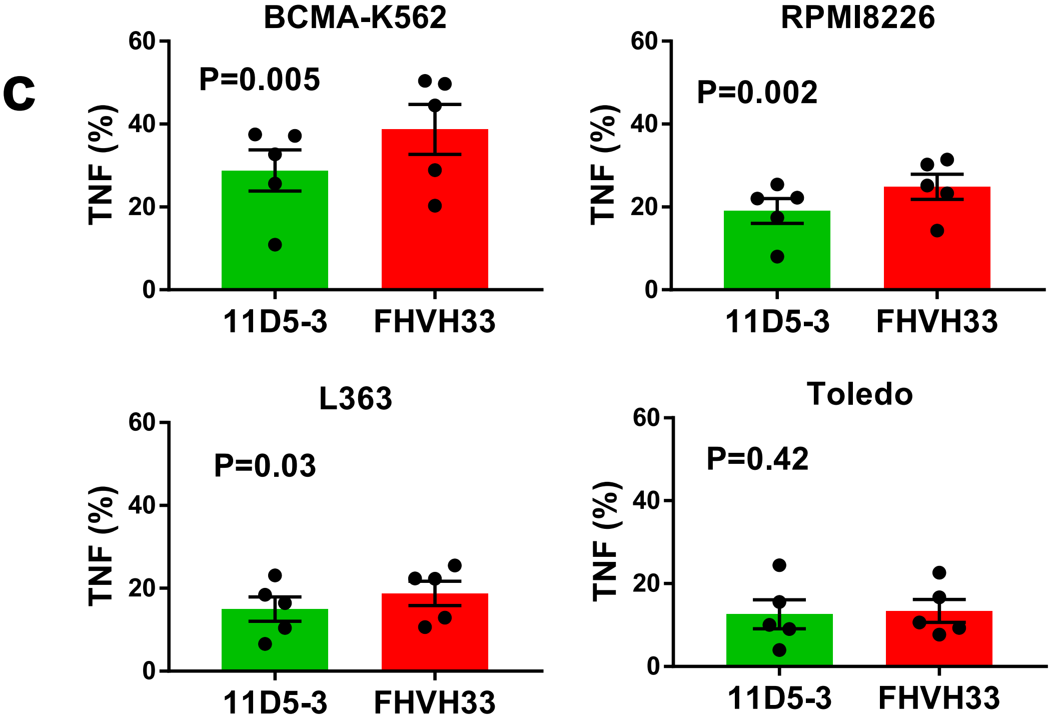

Supplementary Figure 2. FHVH33-CD8BBZ T cells produce more TNF than 11D5-3-CD8BBZ T cells when co-cultured with BCMA⁺ cell lines. (a) BCMA expression was quantified by flow cytometry with a PE anti-BCMA antibody. (b) To quantify the relative BCMA expression level (reported as antibody binding capacity, ABC) on cells from (a), the geometric mean fluorescence intensity (MFI) of PE anti-BCMA staining was compared against the geometric MFI of PE–labeled beads. (c) The %CAR⁺ cells in anti-BCMA CAR-T cell cultures was normalized by adding autologous untransduced T cells, so that the %CAR⁺ was the same for all CAR T cell cultures tested. Intracellular cytokine staining (ICCS) for TNF was performed on anti-BCMA CAR T cells that were co-cultured for 6 hours with the indicated target cells. Data from graphs represent percent CD3⁺ cells that were TNF⁺. Mean+SEM is shown. Comparisons were tested with paired 2-tailed T tests. P-values from each comparison are provided on the plots; n=5 experiments with T cells from 5 different donors. N.S., not statistically significant. When 11D5-3-CD8BBZ and FHVH33-CD8BBZ were compared by ICCS, there was no statistical difference in the production of IFNγ or IL-2.

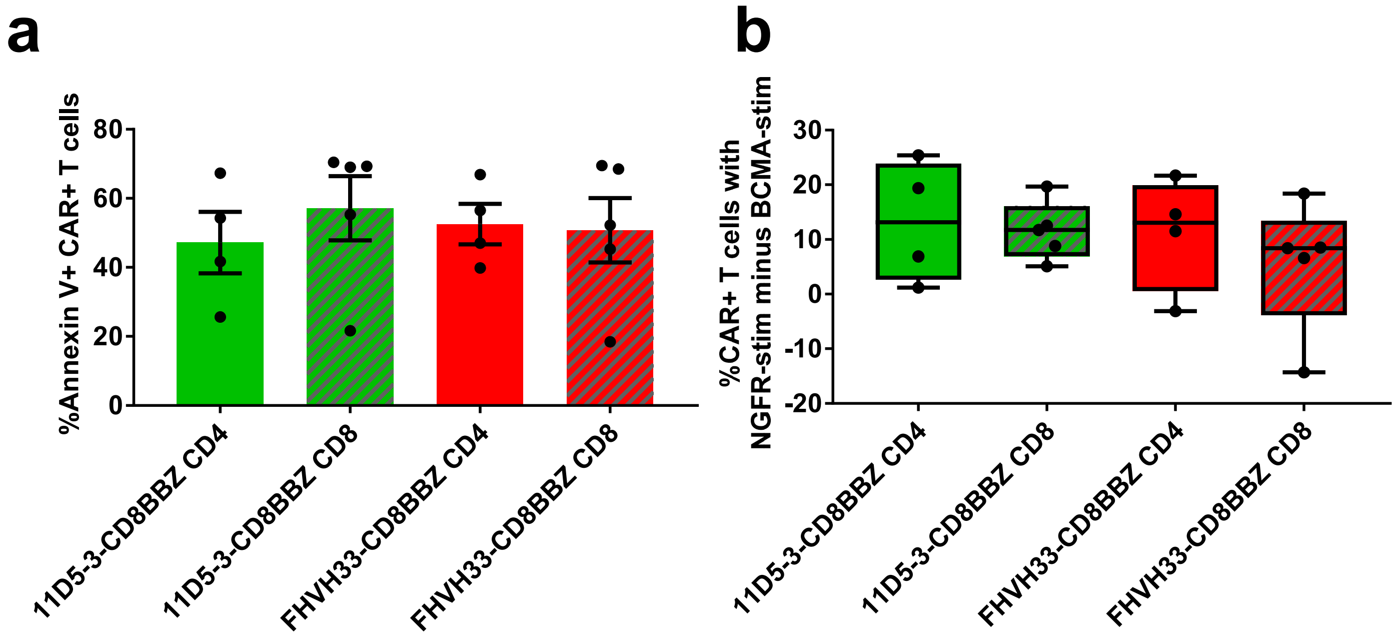

Supplementary Figure 3. Activation-induced cell death of T cells expressing 11D5-3-CD8BBZ versus FHVH33-CD8BBZ. (a) CAR T cells were cultured overnight with BCMA-K562 cells or NGFR-K562 cells and then stained with annexin V and analyzed by flow cytometry. The mean+SEM of the %annexin V^+ CAR^+ T cells after BCMA-K562 stimulation minus the %annexin V^+ CAR^+ T cells after NGFR-K562 stimulation is shown. There was no statistically significant difference in %annexin V^+ cells between 11D5-3-CD8BBZ and FHVH33-CD8BBZ for either $CD4^+$ or $CD8^+$ T cells. Plots of % $annexin^+$ versus $CD4^+$ or $CD8^+$ cells were gated on live CAR^+, $CD3^+$ lymphocytes. (b) For the same cells shown in (a), the %CAR^+ T cells with NGFR-K562 stimulation minus the %CAR^+ T cells with BCMA-K562 stimulation is shown as a measure of BCMA-specific CAR^+ T cell loss or CAR downregulation. Plots of %CAR^+ versus $CD4^+$ or $CD8^+$ cells were gated on live $CD3^+$ lymphocytes. Box and whiskers plots have a horizontal bar at the median and range from the minimum to maximum values. There was not a statistically significant difference between 11D5-3-CD8BBZ and FHVH33-CD8BBZ for either $CD4^+$ or $CD8^+$ T cells. For both (a) and (b) n=5 for $CD8^+$ experiments with T cells from 5 different donors, and n=4 for $CD4^+$ experiments with T cells from 4 different donors; statistics by paired 2-tailed T test.

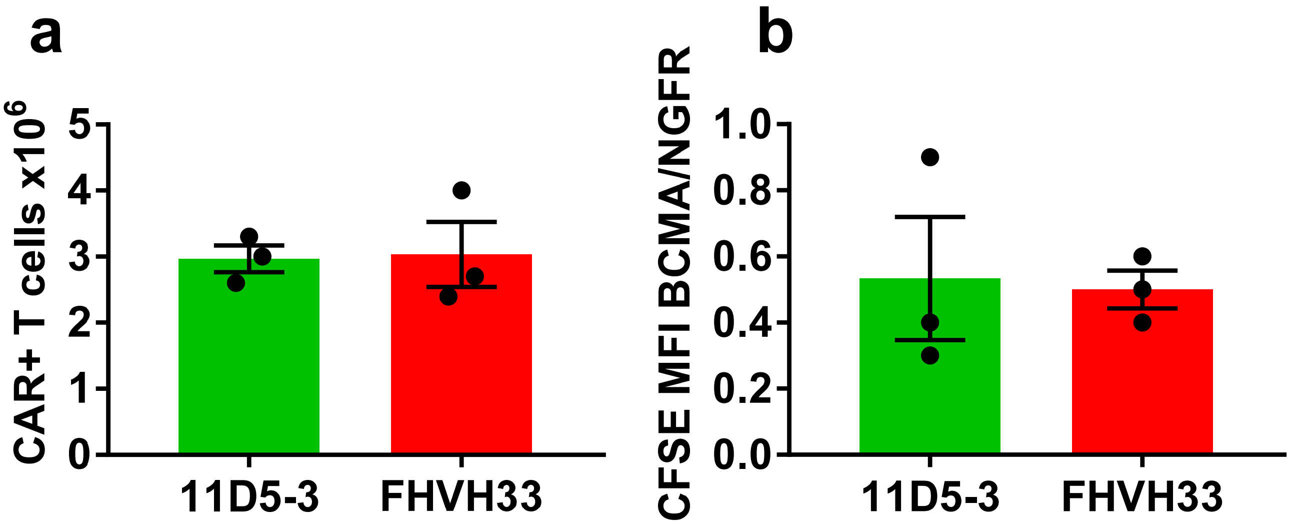

Supplementary Figure 4. Proliferation of T cells expressing 11D5-3-CD8BBZ versus FHVH33-CD8BBZ (a) T cells expressing 11D5-3-CD8BBZ (11D5-3) or FHVH33-CD8BBZ (FHVH33) were labeled with CFSE and cultured with irradiated BCMA-K562 cells or NGFR-K562 cells. Changes in CAR^{+} T cell numbers during the 4-day culture are shown (n=3, experiments with T cells from 3 different donors). All bar graphs in this figure show mean+SEM, and all statistics are paired 2-tailed T tests. (b) BCMA-specific proliferation is represented by the CFSE MFI of T cells stimulated with BCMA-K562 divided by the CFSE MFI of T cells stimulated with NGFR-K562. There were no statistically significant differences in accumulation or proliferation of CAR^{+} cells between 11D5-3-CD8BBZ and FHVH33-CD8BBZ (n=3, experiments with T cells from 3 different donors).

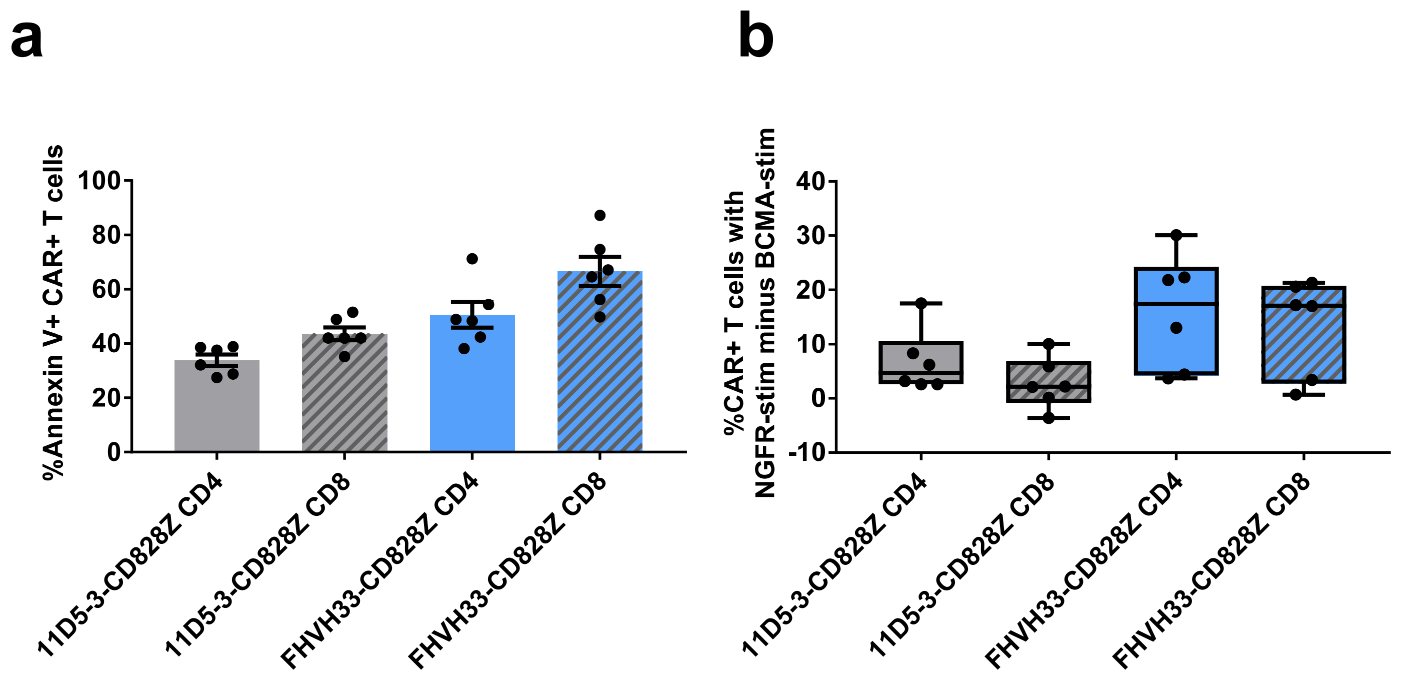

Supplementary Figure 5. Activation-induced cell death of T cells expressing 11D5-3-CD828Z versus FHVH33-CD828Z. (a) CAR T cells were cultured overnight with BCMA-K562 cells or NGFR-K562 cells then stained with annexin V and analyzed by flow cytometry. The mean+SEM of the %annexin V^+ CAR^+ T cells after BCMA-K562 stimulation minus the %annexin V^+ CAR^+ T cells after NGFR-K562 stimulation is shown. Shown are comparisons of %annexin V^+ cells for 11D5-3-CD828Z versus FHVH33-CD828Z; P=0.022 for $CD4^+$ T cells and P=0.004 for $CD8^+$ T cells. Plots of %annexin$^+$ versus $CD4^+$ or $CD8^+$ cells were gated on live CAR^+, $CD3^+$ lymphocytes. (b) For the same cells shown in (a), the %CAR^+ T cells with NGFR-K562 stimulation minus the %CAR^+ T cells with BCMA-K562 stimulation is shown as a measure of BCMA-specific CAR^+ T cell loss or CAR downregulation. Plots of %CAR^+ versus $CD4^+$ or $CD8^+$ cells were gated on live $CD3^+$ lymphocytes. Box and whiskers plots have a horizontal bar at the median and range from the minimum to maximum values. For the comparison of 11D5-3-CD828Z and FHVH33-CD828Z P=0.079 (not significant) for $CD4^+$ T cells; P=0.048 for $CD8^+$ T cells. For both (a) and (b) n=6 experiments with T cells from 6 different donors. For all comparisons; statistics by paired 2-tailed T test, P<0.05 was considered statistically significant.

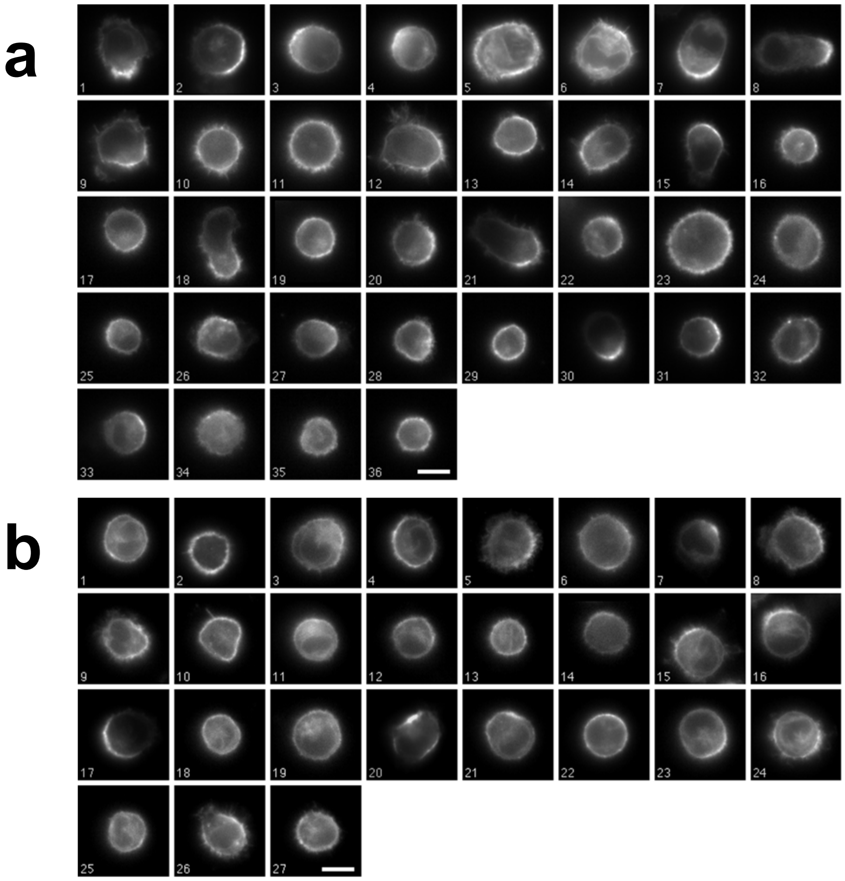

Supplementary Figure 6. Lack of obvious aggregation of either 11D5-3-CD828Z or FHVH33-CD828Z CARs. T cells expressing (a) 11D5-3-CD828Z or (b) FHVH33-CD828Z tagged with the photoswitchable fluorescent protein, Dronpa, were imaged using 488 nm excitation. The fluorescence emission was collected through a longpass 505 filter and an image splitter configured with a polarization beamsplitter. The two orthogonal polarizations were detected on two halves of the camera. The images displayed here represent the parallel fluorescence signal associated with the Dronpa labeled CAR molecules. Scale bars are 5 μm and apply to all images. The total fluorescence signals from the cells varied over an approximately 8-fold range, so these images have been contrast enhanced with a linear function to fully display the localizations and distributions of the molecules in all cells. The localization and distribution of the fluorescence signal showed some cell to cell variation for both molecules including enhanced signals in different regions of the plasma membrane. However, we did not observe cells with numerous punctate regions previously associated with aggregated CAR-T molecules (Long et al. Nature Medicine 2015; Reference 43 in manuscript). The main purpose of this experiment was to assess for obvious differences in CAR aggregation between 11D5-3-CD828Z and FHVH33-CD828Z. We did not observe an obvious difference in punctate staining that has been previously associated with CAR aggregation when we compared T cells expressing these CARs. It is possible that our assay is not able to detect small CAR aggregates; therefore, conclusions about formation of small aggregates cannot be made.

a

Patient 1	*Vector Copy Number/Cell*
MSGV1-FHVH33-CD828Z	2.39
MSGV1-FHVH33-CD8BBZ	2.22
Patient 2	
MSGV1-FHVH33-CD828Z	2.36
MSGV1-FHVH33-CD8BBZ	2.24
Patient 3	
MSGV1-FHVH33-CD828Z	3.11
MSGV1-FHVH33-CD8BBZ	2.67

b

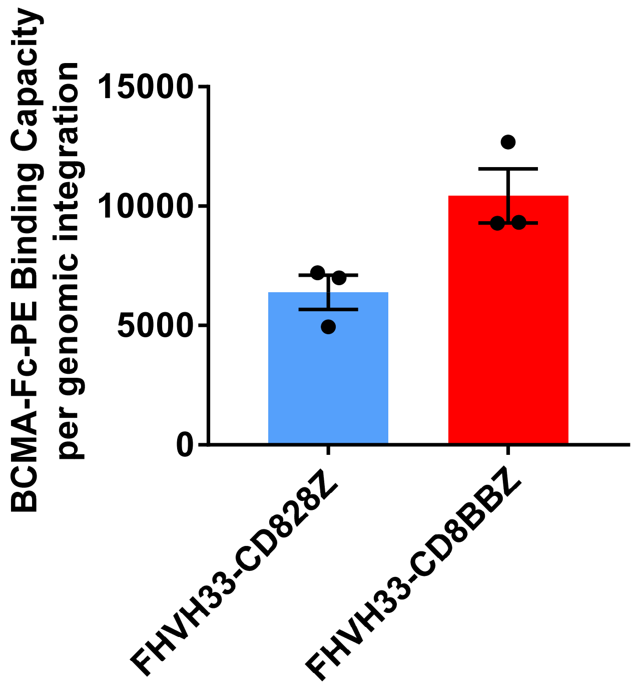

Supplementary Figure 7. Vector copy number and CAR molecule expression analysis of FHVH33 CAR T cells. FHVH33 CAR T cells were sorted for $CD3^{+}$BCMA-Fc-PE^{+} by flow cytometry. (a) Vector copy number was determined for sorted FHVH33 CAR T cells by qPCR using an assay designed against the transgene segment of the MSGV1 γ-retroviral vector. A qPCR assay against human RNaseP was used to normalize γ-retroviral transgene insertions to cell number. Vector copy number/cell was calculated for three sorted donor CAR T cell cultures. (b) To quantify CAR expression, BCMA-Fc-PE binding capacity, was calculated by determining the geometric mean fluorescence intensity (MFI) of BCMA-Fc-PE staining on sorted CAR T cells and comparing geometric MFI against BD Quantibrite PE beads. For sorted CAR T cell cultures from 3 different donors, BCMA-Fc-PE binding capacity per genomic integration was calculated by dividing BCMA-Fc-PE binding capacity by vector copy number/cell. Mean+SEM is shown. For the comparison between FHVH33-CD828Z and FHVH33-CD8BBZ, P = 0.06 (not statistically significant) by paired 2-tailed paired T test.

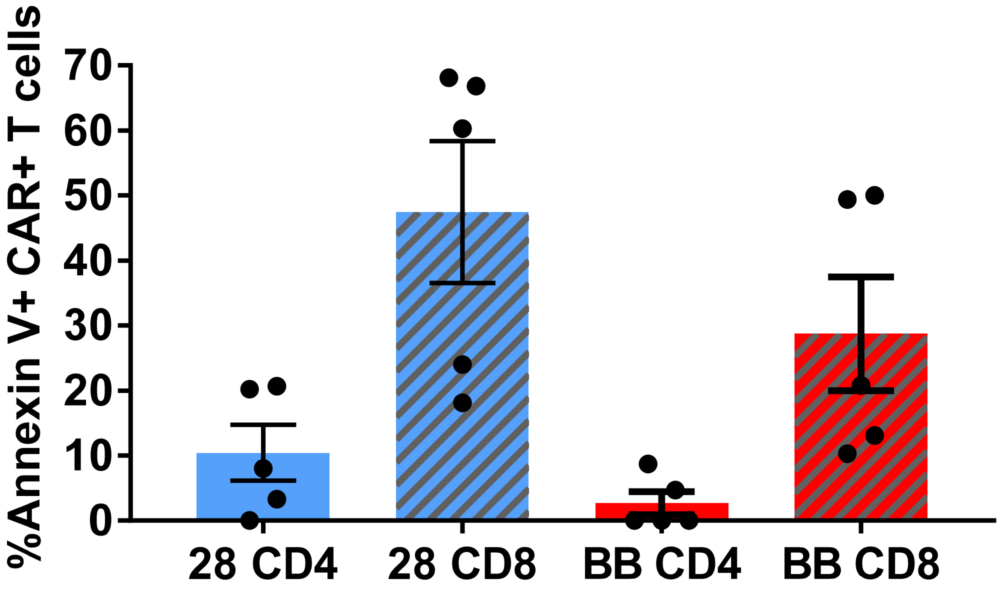

Supplementary Figure 8. Activation-induced cell death of FHVH33 CAR T cells with RPMI8226 target cells. CAR T cells were cultured overnight with RPMI8226 cells and then stained with annexin V and analyzed by flow cytometry. The mean+SEM of the %annexin V^+ CAR^+ T cells after RPMI8226 stimulation minus the %annexin V^+ CAR^+ T cells after NGFR-K562 stimulation is shown. The percent annexin V^+ cells trended toward higher levels for FHVH33-CD828Z (28) T cells compared with FHVH33-CD8BBZ (BB) T cells for $CD4^+$ T cells (P=0.052) The percent annexin V^+ cells was statistically higher for FHVH33-CD828Z T cells compared with FHVH33-CD8BBZ T cells for $CD8^+$ T cells (P=0.028); n=5. Annexin V^+ events were higher for $CD8^+$ T cells than $CD4^+$ T cells for FHVH33-CD828Z (P=0.007) and for FHVH33-CD8BBZ (P=0.022). Statistics were by paired 2-tailed T tests; n=5 different donors. The mean CD4/CD8 $Annexin^+$ ratio was 0.18 for CD28 versus 0.05 for 4-1BB (n=5, P=0.025). Plots of % $annexin^+$ versus $CD4^+$ or $CD8^+$ cells were gated on live CAR^+, $CD3^+$ lymphocytes. All statistics in this figure were by paired 2-tailed T tests. P<0.05 was considered statistically significant.

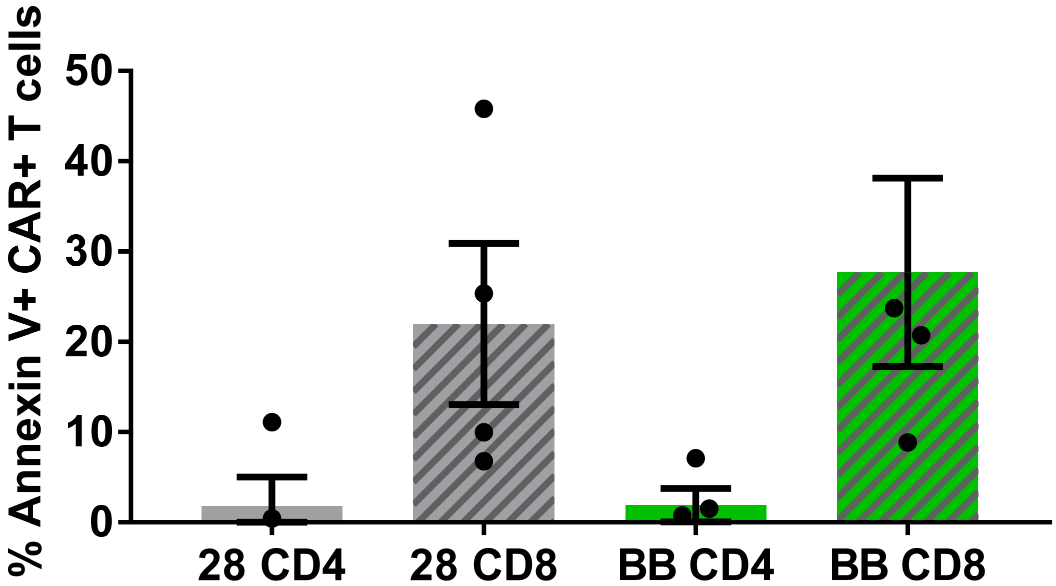

Supplementary Figure 9. Activation-induced cell death of 11D5-3 CAR T cells with RPMI8226 target cells. 11D5-3-CD828Z or 11D5-3-CD8BBZ CAR T cells were cultured overnight with RPMI8226 or NGFR-K562 cells and then stained with annexin V and analyzed by flow cytometry. The graph shows mean+SEM of the %annexin V^+ CAR^+ T cells after RPMI8226 stimulation minus the %annexin V^+ CAR^+ T cells after NGFR-K562 stimulation. There was not a statistical difference in % Annexin V staining between 11D5-3-CD828Z (28) and 11D5-3-CD8BBZ (BB) for $CD4^+$ T cells (P=0.96) or $CD8^+$ T cells (P=0.18). In addition, there was more AICD among $CD8^+$ T cells than $CD4^+$ T cells for 11D5-3-CD828Z (P=0.04), and there was a not statistically-significant trend toward more AICD among $CD8^+$ T cells than $CD4^+$ T cells for 11D5-3-CD8BBZ (P=0.06). Statistics were by paired 2-tailed T tests; P<0.05 was considered statistically significant; n=4 different donors.

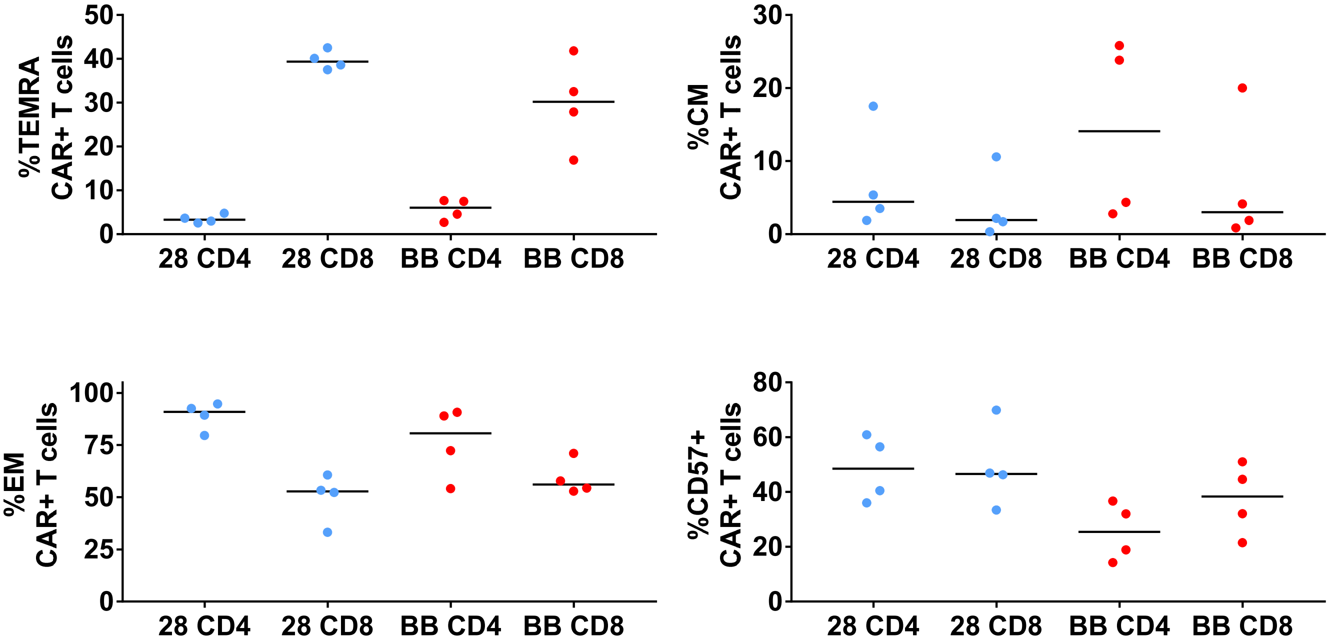

Supplementary Figure 10. Phenotype of T cells expressing FHVH33-CD828Z versus FHVH33-CD8BBZ. T cells expressing FHVH33-CD828Z (28) or FHVH33-CD8BBZ (BB) were analyzed for the expression of CD57 and memory markers at day 15 after vitro culture initiation. Cells were stained for CD57, C-C chemokine receptor type 7 (CCR7), CD45RA, CD3, CD4, CD8, and CAR. CAR^+ T cells were detected by BCMA-Fc-PE staining. T-effector memory (TEMRA) T cells were defined as CCR7-negative, $CD45RA^+$ cells, T effector memory (EM) T cells were defined as CCR7-negative, CD45RA-negative, central memory T cells (CM) were defined as $CCR7^+$, CD45RA-negative T cells. The percentages of cells with the indicated phenotypes double positive for CD3 and CAR, and positive for either CD4 or CD8 are shown for both CARs. The bars on the plots represent medians. Experiments used PBMC of 4 different patients (n=4). When FHVH33-CD828Z and FHVH33-CD8BBZ were compared by 2-tailed, paired T tests, the only statistically significant differences at a level of $P<0.05$ for any of the comparisons between 28 and BB on this figure were the CD57 levels. CD57 expression was lower for FHVH33-CD8BBZ than FHVH33-CD828Z for both $CD4^+$ T cells ($P=0.0014$) and $CD8^+$ T cells ($P=0.0484$).

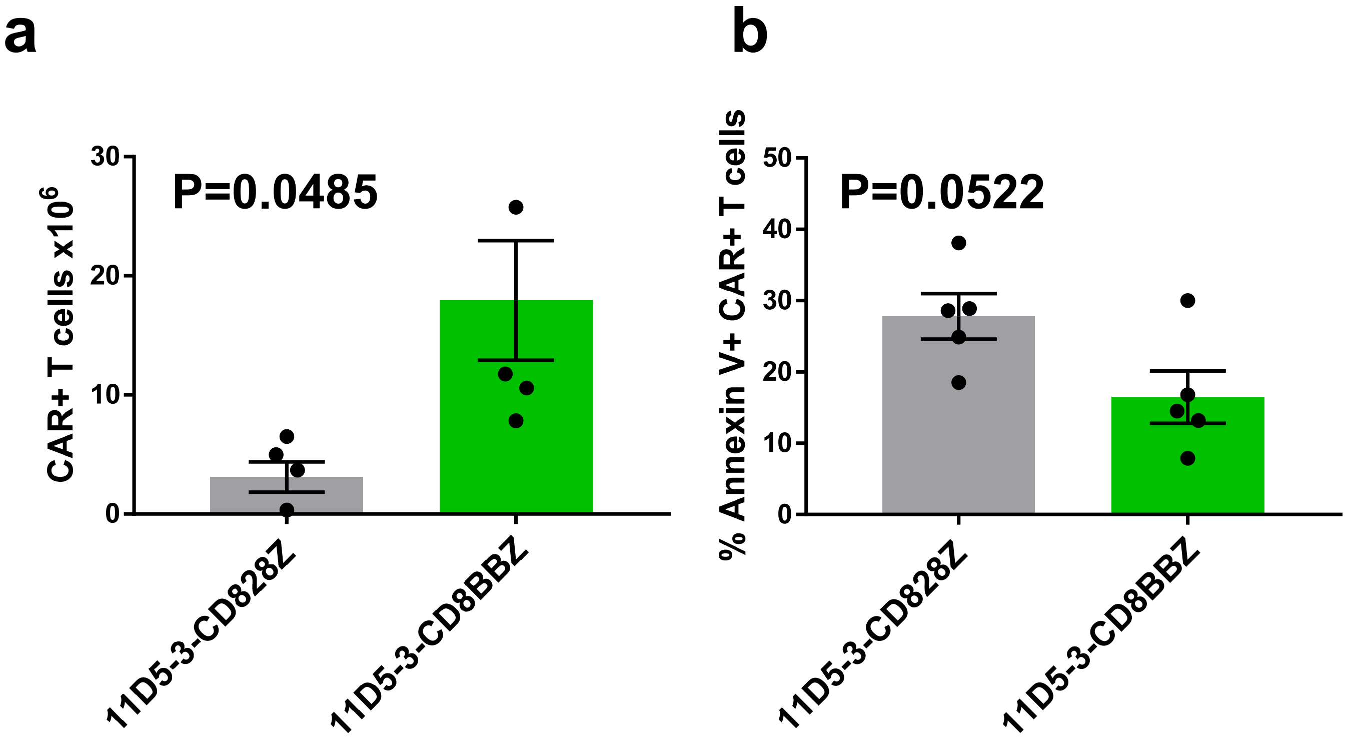

Supplementary Figure 11. Accumulation and apoptosis of 11D5-3 scFv CAR T cells in vitro. Cell accumulation and apoptosis of CAR T cells expressing either 11D5-3-CD828Z or 11D5-3-CD8BBZ were assessed after one in vitro stimulation with BCMA-K562. (a) Anti-BCMA CAR T cells with the 11D5-3 scFv and either a CD28 or 4-1BB costimulatory domain were co-cultured with irradiated BCMA-K562. The change in cell number after 7 days is reported. Mean+SEM is shown. (b) % Annexin $V^{+}CD3^{+}CAR^{+}$ T cells were enumerated by flow cytometry. Mean+SEM is shown. For both (a) and (b), statistical significance was evaluated by using a paired 2-tailed T test. P<0.05 was considered statistically significant. Data are representative of 5 donor T cell cultures.

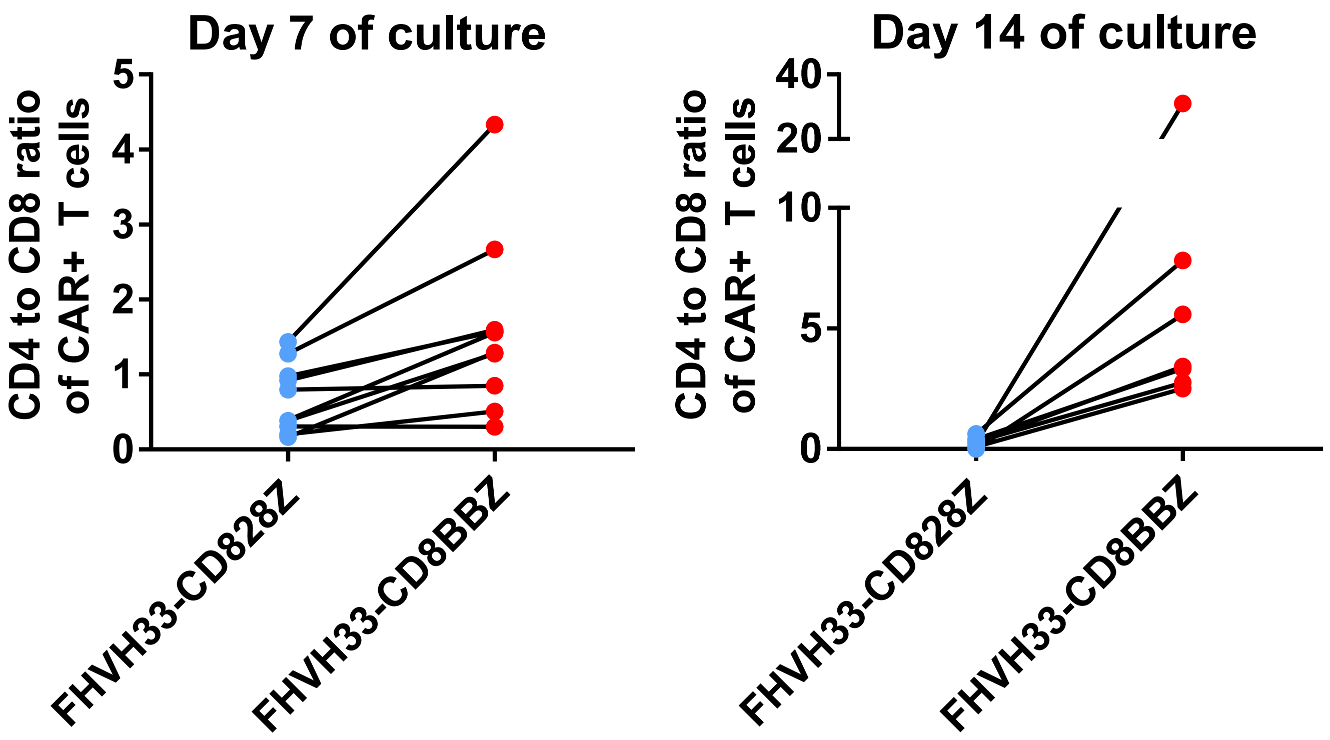

Supplementary Figure 12. Increased CD4:CD8 ratio with T cells expressing FHVH33-CD8BBZ versus FHVH33-CD828Z. T cells were initiated in culture with OKT3 stimulation on day 0. On day 2 of each culture, T cells were transduced. Each experiment included paired cultures of T cells from the same patient that were transduced with either FHVH33-CD828Z or FHVH33-CD8BBZ. Flow cytometry was performed on either day 7 or day 14 of culture for CD3, CD8, CD4, and BCMA-Fc-PE. Cultures included 300 IU/mL of IL-2. The CD4 to CD8 ratio was higher for FHVH33-CD8BBZ than FHVH33-CD828Z at both day 7 and day 14. P=0.0039 and n=10 (T cell cultures from 10 different donors) for day 7 and P=0.0156 and n=7 (T cell cultures from 7 different donors) for day 14, respectively. Statistical comparison by Wilcoxon matched-pairs signed rank test. P<0.05 was considered statistically significant.

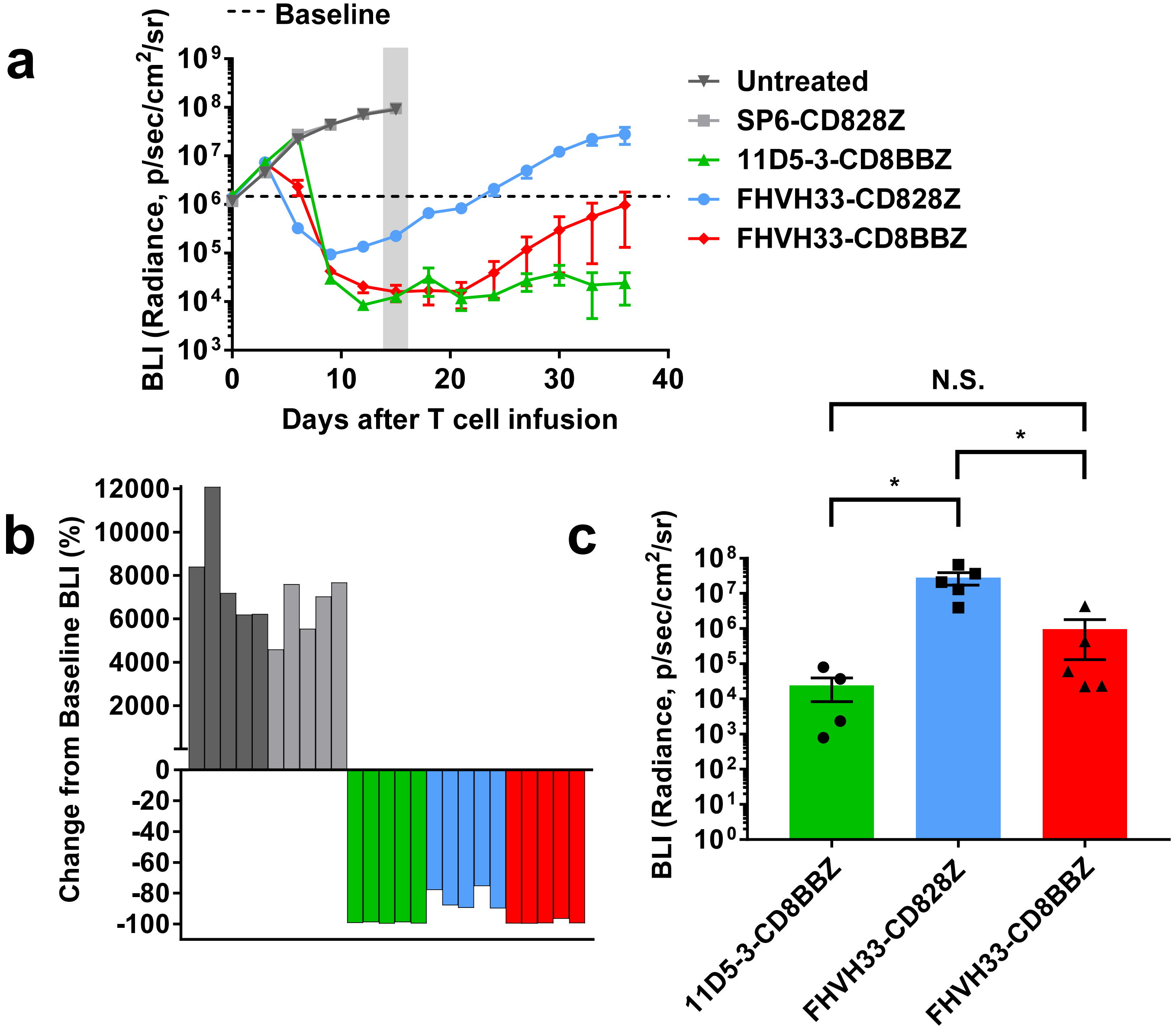

Supplementary Figure 13. CAR T cell studies in disseminated myeloma mouse model. Graphical presentation of MM.1S-Luc tumor bioluminescence (BLI) in Figure 6a. (a) Disseminated MM.1S-luc tumor cells were detected using bioluminescence imaging every three days. Day 0 indicates day of CAR T cell infusion. Shaded box indicates day 15, the last day on which all groups in the study were evaluable for tumors. (b) The change in BLI from day 0 (baseline) to day 15 post-infusion is displayed in a waterfall plot. Mice from untreated and SP6-CD828Z groups were sacrificed at day 15. (c) BLI from 11D5-3-CD8BBZ, FHVH33-CD828Z, and FHVH33-CD8BBZ-treated mice were compared at day 36 post-infusion (n=5, each point represents one mouse). Mean+SEM is shown. The color key in (a) applies to all panels of the figure. For statistical testing, a one-way ANOVA was performed with Tukey's multiple comparisons test. An asterisk (*) indicates a $P<0.05$. N.S. is not significant. For 11D5-3-CD8BBZ vs. FHVH33-CD828Z, $P=0.02$. For 11D5-3-CD8BBZ vs. FHVH33-CD8BBZ, $P=0.99$, N.S. For FHVH33-CD828Z vs. FHVH33-CD8BBZ, $P=0.025$.

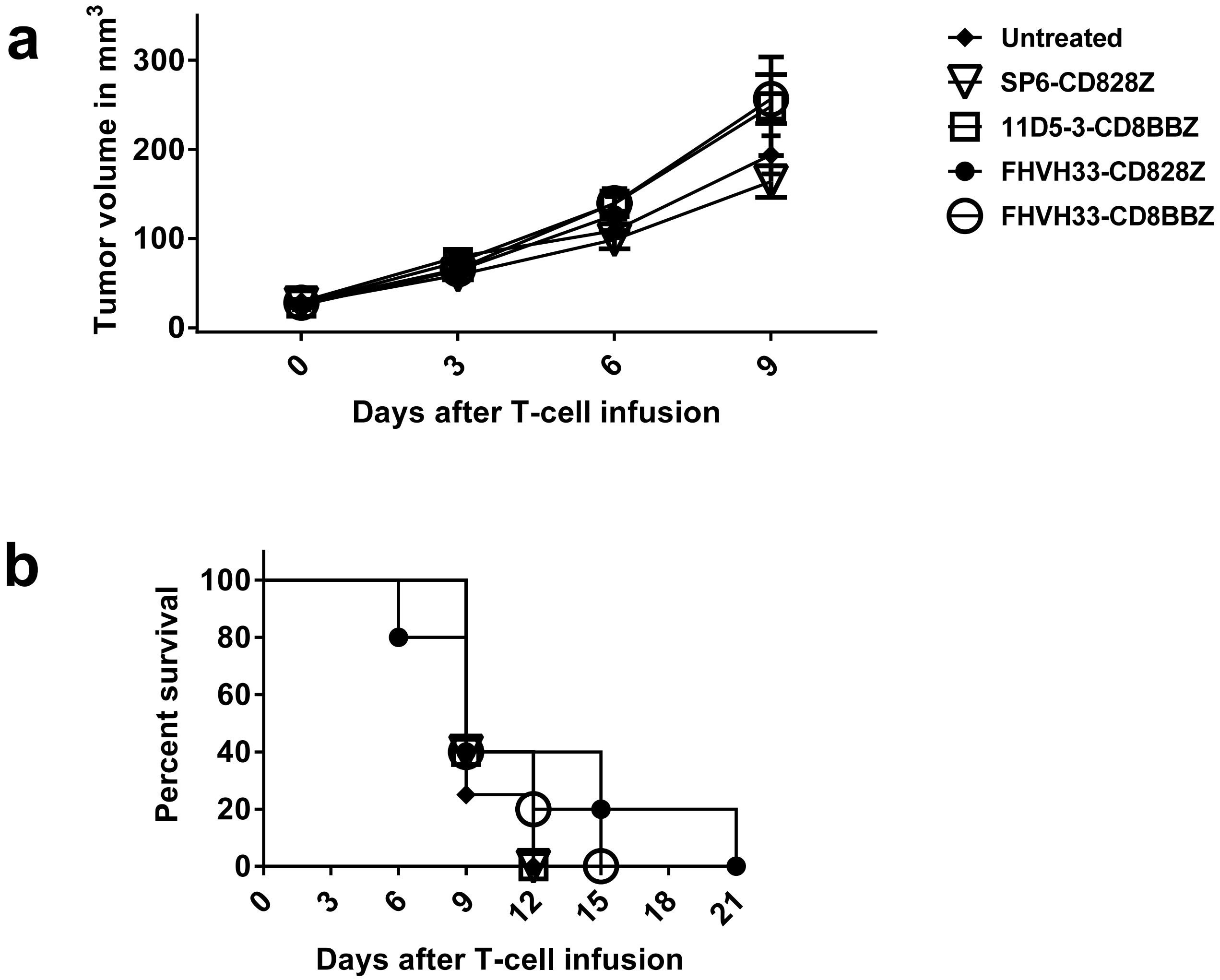

Supplementary Figure 14. Treatment of solid RPMI8226 tumors with 0.5x10^6 CAR T cells/mouse. RPMI8226 cells were injected intradermally into NSG mice. After palpable tumors were established, mice were injected with 0.5x10^6 T cells expressing the indicated CARs. (a) Mean tumor volume of 5 mice/group and (b) Kaplan-Meier plots of survival of the same mice are displayed above. There was no evident anti-tumor activity by the CAR T cells at the 0.5x10^6 T cells/mouse dose.

a

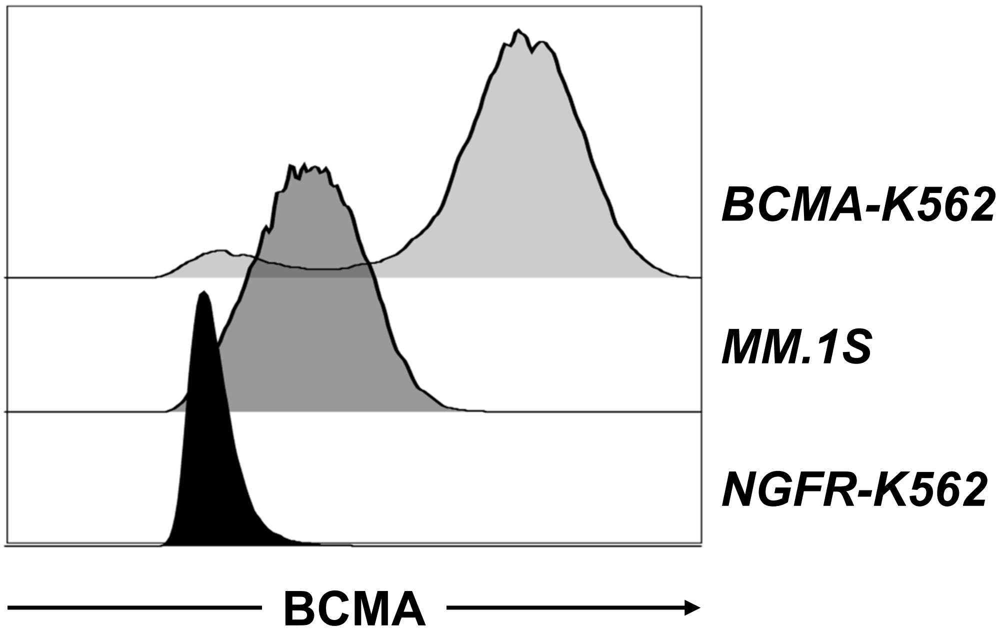

b

	BCMA ABC/Isotype ABC
BCMA-K562	35
MM.1S	11
NGFR-K562	1

Supplementary Figure 15. BCMA expression level on BCMA-K562 cells versus MM.1S cells. (a) BCMA expression was quantified using a PE-conjugated BCMA antibody. (b) Geometric mean fluorescence intensity of BCMA staining was determined by flow cytometry and BCMA antibody binding capacity (ABC) was determined using BD Quantibrite PE beads. The ratio of BCMA ABC to isotype ABC for each cell line was determined.

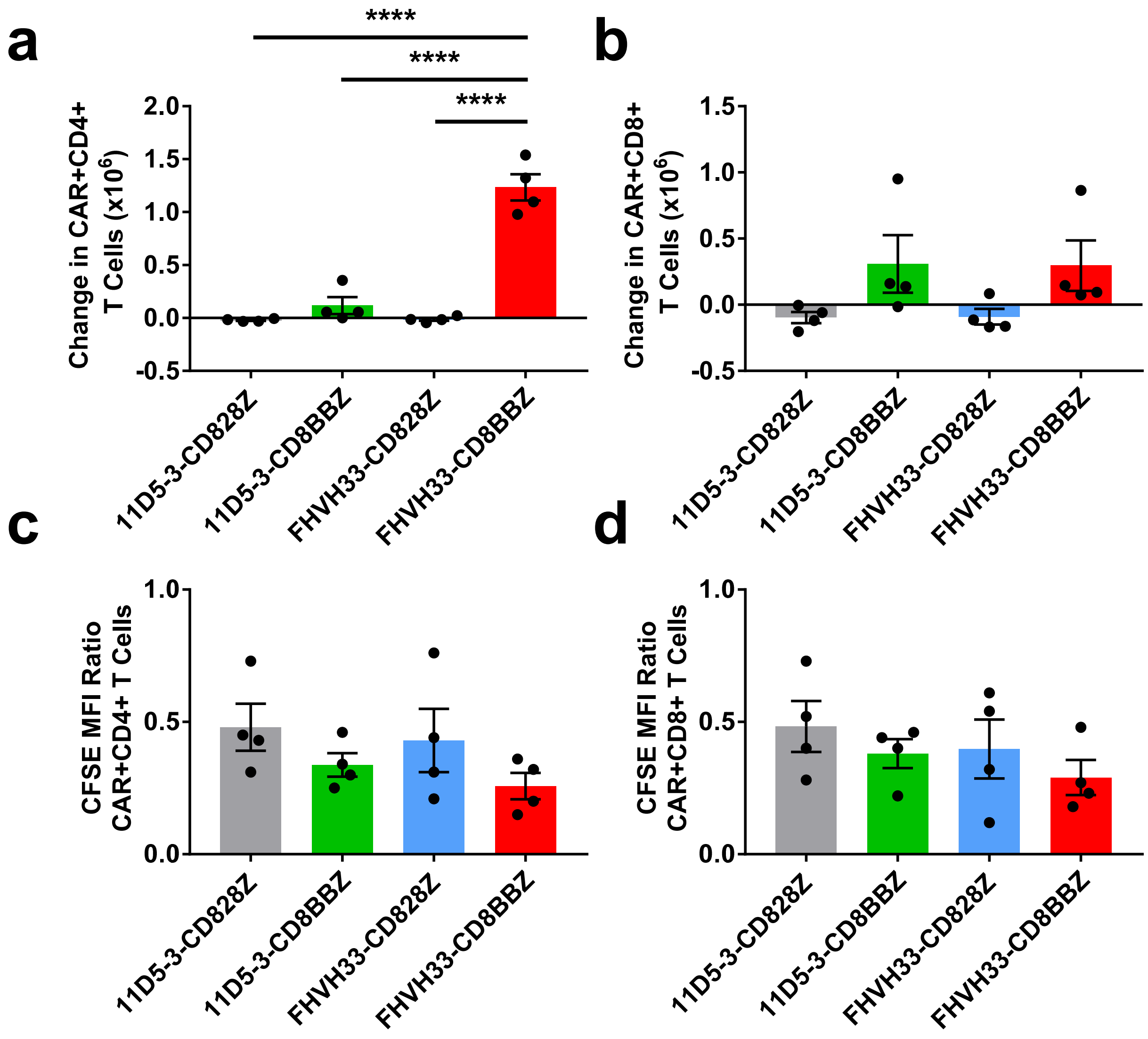

Supplementary Figure 16. Proliferation of anti-BCMA CAR T cells in response to MM.1S cells. (a-b) Anti-BCMA CAR T cells were labeled with CFSE and co-cultured with MM.1S ($BCMA^+$) cells for four days. T cells were analyzed by flow cytometry and the absolute numbers of (a) $CD3^+CAR^+CD4^+$, and (b) $CD3^+CAR^+CD8^+$ T cells were determined. The change in CAR T cell numbers was calculated by subtracting the initial absolute number of CAR^+ T cells from the absolute cell number of CAR^+ T cells at the end of the co-culture. FHVH33-CD8BBZ $CD4^+$ T cells accumulated more than T cells expressing other CARs. (c-d) The CFSE median fluorescence intensity (MFI) of (c) $CD3^+CAR^+CD4^+$, and (d) $CD3^+CAR^+CD8^+$ T cells was quantified after a four-day co-culture. CFSE MFI ratios were calculated by dividing the CFSE MFI values of T cells co-cultured with MM.1S by the CFSE MFI values of T cells co-cultured with NGFR-K562. Data represent experiments from four independent T cell cultures (n=4). For each panel of this figure, data was analyzed with a one-way repeated measures ANOVA and a Tukey's multiple comparisons test; **** $P < 0.01$. Lack of asterisks indicates lack of statistical significance. The only statistically significant differences were in panel (a).

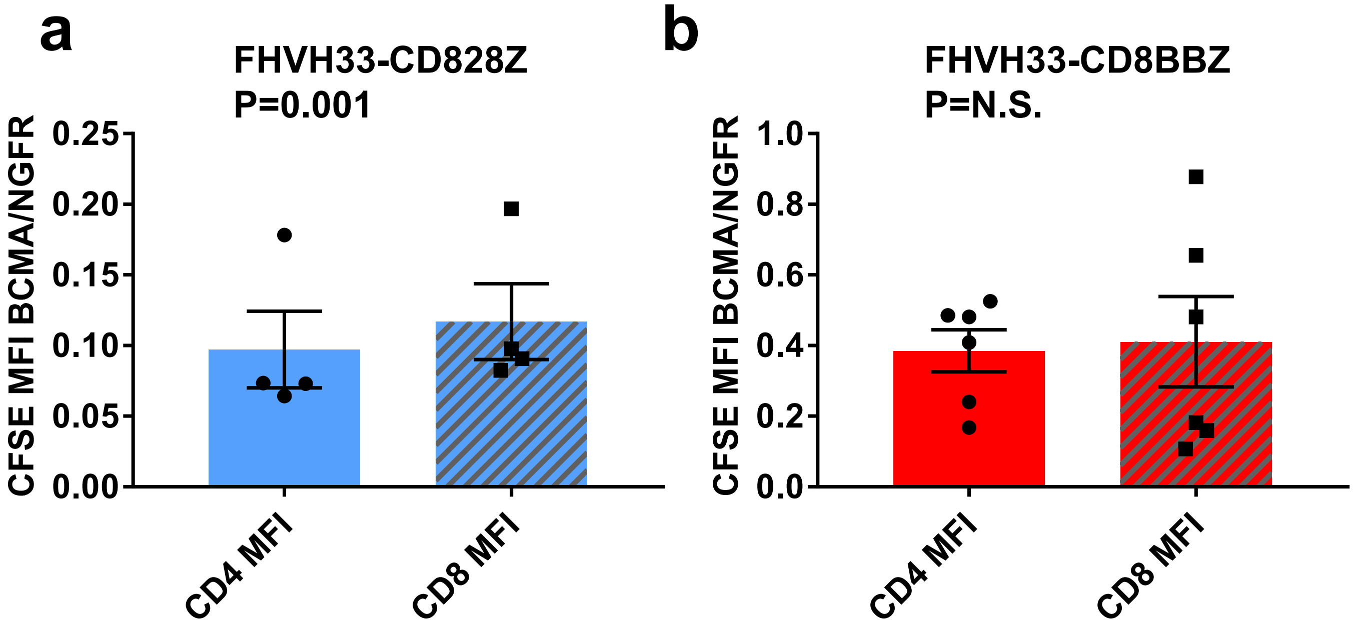

Supplementary Figure 17. CD4⁺ versus CD8⁺ T cell proliferation of FHVH33-CD828Z and FHVH33-CD8BBZ CAR T cells. (a) FHVH33-CD828Z or (b) FHVH33-CD8BBZ T cells were labeled with CFSE and cultured with either irradiated BCMA-K562 cells or NGFR-K562 cells. BCMA-specific proliferation is represented by the CFSE median fluorescence intensity (MFI) of T cells stimulated with BCMA-K562 divided by the CFSE MFI of T cells stimulated with NGFR-K562. Flow plots for determining CFSE MFI were gated on $CD4^+$ or $CD8^+$ live CAR^+CD3^+ lymphocytes. All bar graphs in this figure show mean+SEM, and all statistics are paired 2-tailed T tests; n=4 (experiments with T cells from 4 different donors) for FHVH33-CD828Z; n=6 (experiments with T cells from 6 different donors) for FHVH33-CD8BBZ. For FHVH33-CD828Z T cells, $CD4^+$ T cells proliferated slightly more than $CD8^+$ T cells. For FHVH33-CD8BBZ, proliferation was not different for $CD4^+$ versus $CD8^+$ T cells.

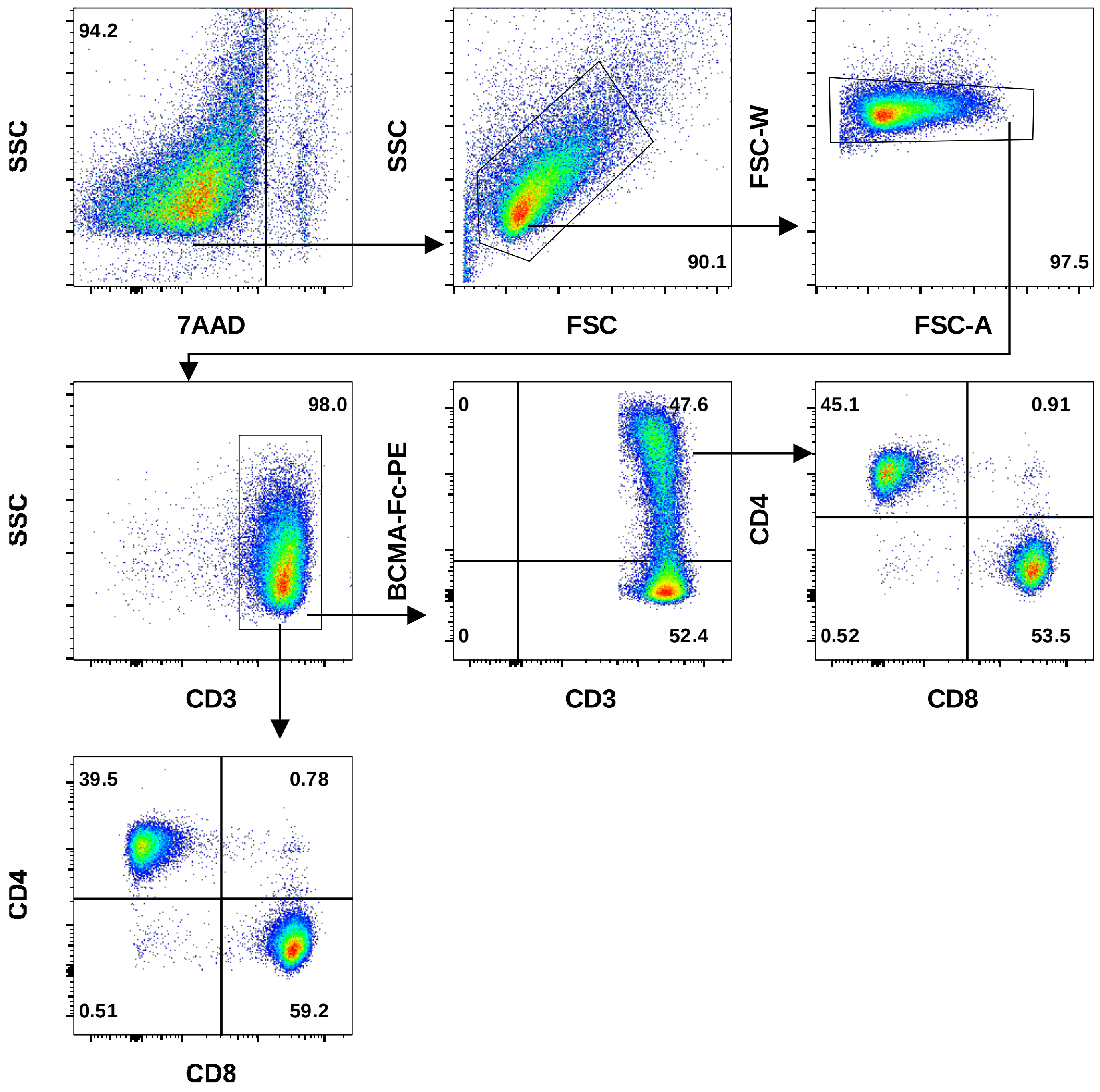

Supplementary Figure 18. Gating strategy for anti-BCMA CAR T cell flow cytometry. All flow experiments with anti-BCMA CAR T cells were analyzed using the following strategy. T cell cultures were gated on 7AAD-negative single lymphocytes that were $CD3^{+}CAR$ (BCMA-Fc-PE)$^{+}$. Analysis of CD4, CD8, annexin V, and memory markers was performed on only the CAR^{+} population. Analysis of cytokine-secreting T cells (for intracellular flow experiments) or degranulating T cells (for CD107a experiments) was performed on total $CD3^{+}CD4^{+}$ or $CD3^{+}CD8^{+}$ populations due to CAR downregulation following BCMA recognition.

	BCMA-K562	RPMI-8226	NGFR-K562	CCRF-CEM	T cells Only	%CAR+
**Untransduced**	149.7	212.0	151.4	46.0	15.6	0
**FHVH74-CD8BBZ**	39295.7	38815.4	295.2	120.5	68.7	11.5
**FHVH32-CD8BBZ**	66299.6	39025.3	290.1	177.3	155.2	54.2
**FHVH33-CD8BBZ**	63188.1	39618.4	266.8	144.6	108.1	61.1
**FHVH93-CD8BBZ**	47452.1	32720.8	199.0	157.5	140.8	58.8

Supplementary Table 1. Anti-BCMA FHVH CAR T cells produce Interferonγ (IFNγ) against BCMA$^+$ cell lines. Untransduced T cells or T cells expressing one of the CARs listed in the far left column were cultured overnight with one of the target cells listed in the top row. An IFNγ ELISA was performed on the culture supernatants. BCMA-K562 and RPMI8226 are BCMA$^+$. NGFR-K562 and CCRF-CEM are BCMA-negative. The % of T cells expressing each CAR is listed in the far right column (%CAR$^+$). Aside from the %CAR$^+$ column, all values in the table are pg/mL of IFNγ. Data shown are representative of two independent experiments using T cells derived from 2 different donors (n = 2).

	BCMA-K562			
**ng/mL sBCMA**	**150**	**50**	**25**	**0**
FHVH33-CD828Z	34821	38582	37225	35576
FHVH33-CD8BBZ	70008	63175	61903	60361

	RPMI8226			
**ng/mL sBCMA**	**150**	**50**	**25**	**0**
FHVH33-CD828Z	14870	17680	16949	17583
FHVH33-CD8BBZ	30838	34304	30650	31937

	T cells only			
**ng/mL sBCMA**	**150**	**50**	**25**	**0**
FHVH33-CD828Z	24	18	21	22
FHVH33-CD8BBZ	117	114	125	122

Supplementary Table 2. Effect of solubilized BCMA on FHVH33 CAR T cells. T cells expressing the anti-BCMA CAR T cells listed in the far left column were co-cultured overnight with the BCMA-positive target cells BCMA-K562 or RPMI8226. The CAR T cells were also cultured without other cells (T cells only). Soluble BCMA protein was added to cultures in the concentrations indicated, 150, 50, 25, or 0 ng/mL. Culture supernatants were analyzed for IFNγ production by ELISA. The values in the bottom 2 rows are IFNγ in pg/mL. Data shown are representative from two independent experiments using T cells derived from 2 different donors. The solubilized BCMA neither blocked recognition of BCMA$^+$ target cells nor caused nonspecific CAR T cell activation. sBCMA, soluble BCMA.
